# Immunomagnetic Capture of Faecalibacterium prausnitzii Selectively Modifies the Fecal Microbiota and Its Immunomodulatory Profile

**DOI:** 10.1128/spectrum.01817-22

**Published:** 2023-01-04

**Authors:** Raquel Marcos-Fernández, Sabino Riestra, Rebeca Alonso-Arias, Lorena Ruiz, Borja Sánchez, Abelardo Margolles

**Affiliations:** a Departamento de Microbiología y Bioquímica, Instituto de Productos Lácteos de Asturias, Consejo Superior de Investigaciones Científicas (IPLA-CSIC), Villaviciosa, Asturias, Spain; b Instituto de Investigación Sanitaria del Principado de Asturias (ISPA), Oviedo, Asturias, Spain; c Departamento de Gastroenterología, Unidad de Enfermedad Inflamatoria Intestinal, Hospital Universitario Central de Asturias (HUCA), Oviedo, Asturias, Spain; d Departamento de Inmunología, Hospital Universitario Central de Asturias (HUCA), Oviedo, Asturias, Spain; e Department of Cardiac Pathology, Health Research Institute of the Principality of Asturias (ISPA), Oviedo, Asturias, Spain; Indian Institute of Science Bangalore

**Keywords:** *Faecalibactarium prausnitzii*, Crohn disease, flow cytometry, immunomagnetic separation, immunomodulation, gut microbiota, immunoseparation

## Abstract

*Faecalibacterium* represents one of the most abundant bacterial groups in the human intestinal microbiota of healthy adults and can represent more than 10% of the total bacterial population, Faecalibacterium prausnitzii being the only recognized species up to the past year. Reduction in the abundance of *F. prausnitzii* in the human gut has been linked to several human disorders, such as Crohn’s disease. In this study, we developed a strategy to modify the relative abundance of *F. prausnitzii* in fecal microbiotas as a means of evaluating its contribution to the immunomodulatory effect of intestinal microbiotas with different *F. prausnitzii* contents using a peripheral blood mononuclear cell (PBMC) model. We used a polyclonal antibody against the surface of *F. prausnitzii* M21 to capture the bacterium from synthetic and human fecal microbiotas using immunoseparation techniques. As a proof-of-principle study, the levels of immunomodulation exerted by microbiotas of healthy donors (HDs) with different relative abundances of *F. prausnitzii,* achieved with the above-mentioned immunoseparation technique, were evaluated in a PBMC model. For this purpose, PBMCs were cocultivated with the modified microbiotas or a pure culture of *F. prausnitzii* and, subsequently, the microbiota of Crohn’s donors was added to the coculture. The cytokine concentration was determined, showing that our experimental model supports the anti-inflammatory effects of this bacterium.

**IMPORTANCE** There is increasing interest in deciphering the contribution of gut microbiota species to health and disease amelioration. The approach proposed herein provides a novel and affordable strategy to probe deeply into microbiota-host interactions by strategically modifying the relative abundance of specific gut microbes, hence facilitating the study of their contribution to a given trait of the microbiota.

## INTRODUCTION

The *Faecalibacterium* genus encompasses a group of Gram-stain-positive, non-spore-forming, acetate-consuming and butyrate-producing, extremely oxygen-sensitive (EOS) members of the phylum *Firmicutes*. The type strain of the Faecalibacterium prausnitzii species, the only species recognized within this genus up to 2021, was originally isolated from human feces in the 1970s and classified as Fusobacterium prausnitzii (1). Two decades later, with the aid of 16S rRNA sequence information, it was reassigned to the Clostridium leptum group (clostridial cluster IV) (2), and in 2002, it was classified as a separate genus named *Faecalibacterium* (family *Ruminococcaceae*) by Duncan et al. (3). Despite a large number of bacteria, archaea, viruses, and unicellular eukaryotes the inhabit the human body, only a few bacterial genera, such as *Bacteroides*, *Bifidobacterium*, and *Faecalibacterium*, predominate in the gut microbiome (4). High-throughput sequencing of 16S rRNA libraries and metagenomic analysis of fecal DNA revealed that the genus *Faecalibacterium* represents one of the most abundant bacterial groups in the healthy human gut, where it can represent more than 10% of the total bacterial population (5–9). *F. prausnitzii* was demonstrated to play an important role in providing energy to the colonocytes and maintaining intestinal health, being considered one of the most important butyrate-producing bacteria in the human gut (10). At the same time, decreased *F. prausnitzii* levels are frequently observed in various diseases with associated intestinal inflammatory processes, such as inflammatory bowel disease (IBD), type 2 diabetes, and colorectal cancer (9, 11–13). In view of these facts, increasing research has focused on this particular gut bacterium, which has been proposed as a potential next-generation probiotic (14). This has accelerated research into this bacterial group, and expanding comparative genomic investigation has led to a proposed separation of former *F. prausnitzii* species into different *Faecalibacterium* species, including *F. longum*, *F. butyricigenerans*, *F. prausnitzii*, *F. duncaniae; F. hattorii*, and *F. gallinarum*, together with a number of still unassigned *Faecalibacterium* species (15–17). Thus, a word of caution should be given as most literature up to the past year referred exclusively to *F. prausnitzii* species, while several of the strains have been recently reclassified into novel separate species. It is also worth noting that recent comparative analyses that motivated the separation of the former *F. prausnitzii* into several distinct novel species also evidenced that prior work based on quantitative PCR (qPCR) detection and quantification of *Faecalibacterium* cells may be biased as most primers used in literature are not equally efficient to detect all the currently recognized *Faecalibacterium* species (18). Therefore, some research results conducted prior to the species reclassification may be biased and attribution of traits of interest and biomarker assignments up to the species level may require revision. For simplification, and unless otherwise specified, in this article *F. prausnitzii* generally refers to the former *Faecalibacterium* species group and may include strains now reclassified into novel species.

Inflammatory bowel disease (IBD) is a group of disorders characterized by a chronic and relapsing inflammation of the gastrointestinal tract and is experiencing a rapid increase in prevalence (19). Growing evidence has led to propose that genetic and environmental factors play a critical role in the pathogenesis of different manifestations of IBD, including Crohn’s disease (CD) (20, 21). Among the factors influencing CD, the intestinal microbiota has received increased attention in the last few years. In particular, low abundance of *Faecalibacterium* has been linked with active CD (11, 22, 23). Other than butyrate production (which can reduce intestinal mucosa inflammation and is the main energy source for the colonocytes), additional anti-inflammatory properties have been attributed to *Faecalibacterium* cells (9, 11, 24). Both whole cells and supernatant fractions of this bacterial group were able to reduce severity of acute (11, 25), chronic (24), and low-grade (26) inflammation in murine models. This has been attributed to an enhancement of intestinal barrier function related to the promotion of the expression of certain tight junction proteins (27). *Faecalibacterium* also influences gut physiology through the production of mucus O-glycans and may help to maintain suitable proportions of different secretory cell types in the intestinal epithelium, as evidenced in rodent studies (28).

**FIG 1:**
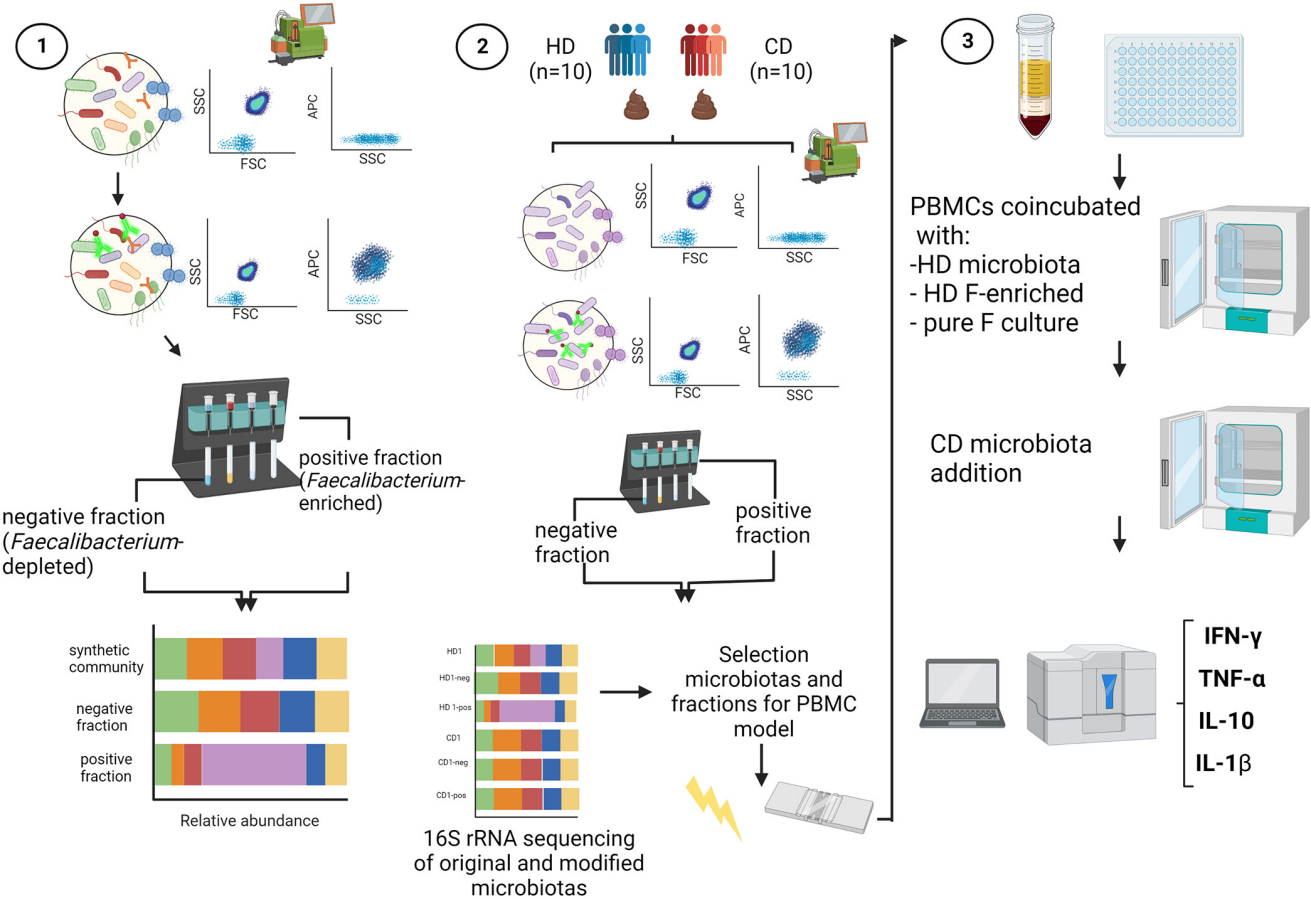
Schematic diagram representing the workflow of the different experiments conducted on this research. (Step 1) First, an antibody targeting *F. prausnitzii* M21 cellular envelopes was generated, and its utility to obtain *Faecalibacterium-*enriched and *Faecalibacterium*-depleted fractions from a synthetic microbial community was evaluated. (Step 2) Subsequently, the targeted microbiota procedure was applied to real fecal microbiotas obtained from healthy donors (HD) and Crohn’s disease (CD) patients. The 16S rRNA analysis of original and modified microbiotas enabled selection of fractions to be analyzed in a coculture model with PBMCs. (Step 3) A coculture model of a selection of modified microbiotas with PBMCs was used to determine whether preexposure of PBMCs to *Faecalibacterium*-enriched microbiotas could counteract the inflammatory profile induced by representative microbiotas from CD patients.

The present work makes use of cell immunoseparation techniques coupled to flow cytometry (FC) to shed, *in vitro*, some light on the cross talk dialogue between *Faecalibacterium* cells present in complex fecal microbiota samples and peripheral blood mononuclear cells (PBMCs) in the framework of CD. The study presented in this article has a dual goal. On one hand, we are proposing a novel methodology to probe more deeply into the functions of specific members of the human microbiota by artificially modifying its representation in real fecal microbiotas. On the other hand, our findings contribute to settled scientific evidence about the anti-inflammatory potential of *Faecalibacterium* when present in a complex microbiota since by enriching and depleting specific microbes, we can modulate the immune cell responses induced by fecal microbial populations. A schematic overview of the workflow followed is presented in Fig. 1. Furthermore, our methods can be easily translated to other members of the intestinal microbiota or even to other complex microbial environments.

## RESULTS

### Specificity of the polyclonal antibody against *F. prausnitzii* M21: evaluation by flow cytometry, confocal microscopy, and Western blotting.

**FIG 2:**
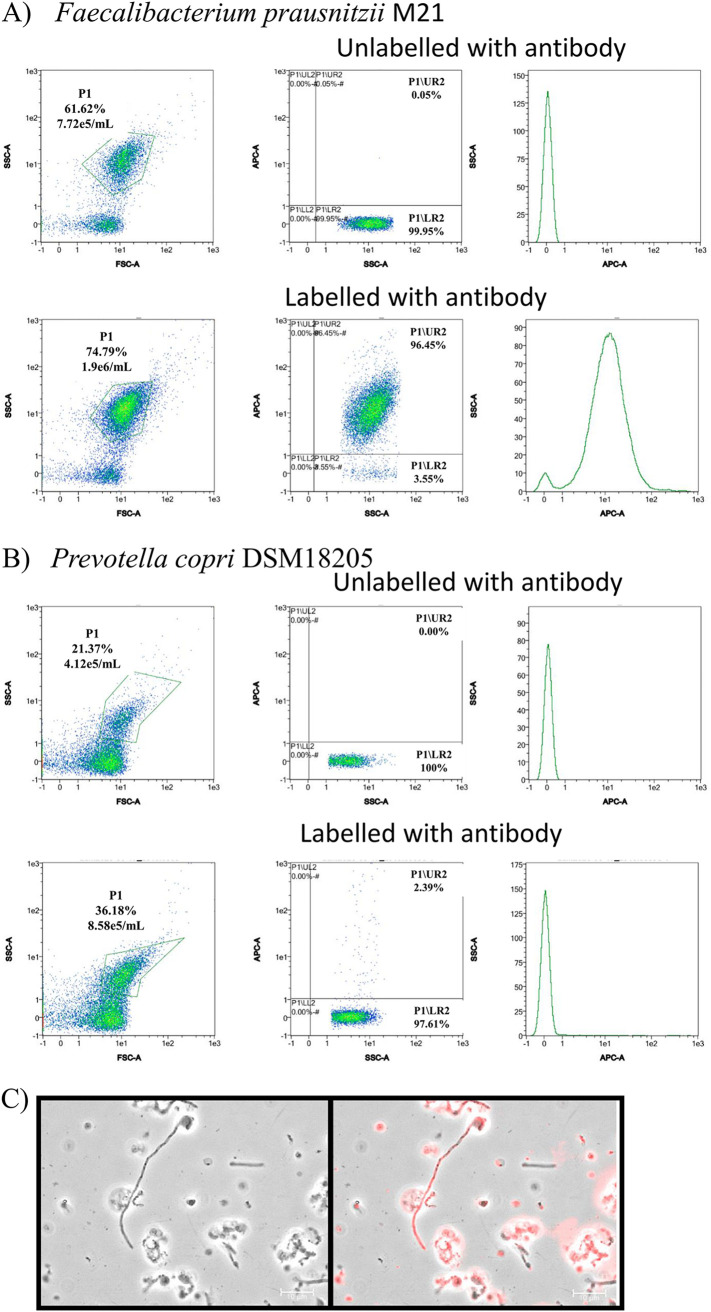
Dispersion diagrams of representative flow cytometry experiments showing the acquisition of *F. prausnittzii* M21 (A) and Prevotella copri (B) labeled (lower panels) and not labeled (upper panels) with the antibody raised against *F. prausnitzii* M21. On each set of experiments, the following plots are presented: SSC versus FSC (left-most panel), highlighting the P1 gate that identifies events corresponding to bacterial cells; APC-A versus SSC (middle panel), which highlights the P1 events displaying fluorescence signal in the APC channel (P1/UR2) and the P1 events not displaying any fluorescence signal (P1/LR2) against the SSC; and the number of events versus APC-A (right panel), which represents the number of events identified at each fluorescence intensity in the APC channel. Immunofluorescence microscopy photography showing the binding of the polyclonal antibody to unlabeled (left picture) and labeled (right picture) *F. prausnitzii* M21 cells is also presented (C). At least triplicate experiments were carried out.

The specificity of the generated antibody was determined by evaluating whether it could recognize and label the target species strain *F. prausnitzii* M21 and whether it could lead to nonspecific labeling of other bacterial species different from *Faecalibacterium*. For this purpose, the target strain and 10 different bacterial species selected as common inhabitants of the human gut and belonging to the most common phyla and families encountered within the gut ecosystem were labeled with the anti-*F. prausnitzii* M21 polyclonal antibody conjugated to the fluorescent molecule allophycocyanin (APC). The labeling efficiency was first evaluated by flow cytometry. Figure 2 illustrates the specificity of the polyclonal antibody in the recognition of *F. prausnitzii* cells, with virtually 96.45% of bacteria labeled after 15 min of incubation. The dispersion diagrams were obtained by separating the bacteria by flow cytometry according to side scatter (SSC), forward scatter (FSC), and their fluorescence in the APC channel (corresponding to the set of filters that specifically detect the fluorescence emitted by APC). Data are displayed as dot plots of SSC versus FSC and APC versus SSC. Three regions were designated P1, LR2, and UR2. P1 includes the bacteria, LR2 included the nonfluorescent bacteria, and UR2 included the fluorescent bacteria. The higher emission of *F. prausnitzii* M21 and the lower emission of other representative gut bacterial species can be easily deduced comparing the dispersion plots of M21 (Fig. 2A) with those of Prevotella copri DSM18205, which presented 2.39% of fluorescent events (Fig. 2B). The percentage of fluorescent events in the dispersion plots of the rest of the bacterial members used for the construction of synthetic microbiotas ranged from 0.23 to 10.69% (see Fig. S2 in the supplemental material).

As polyclonal anti-*F. praustnitzii* antibody bound to M21 cells more efficiently than to the rest of the species, a series of immunostaining microphotographs were performed. Immunofluorescence microscopy showed that the APC-conjugated antibody efficiently bound the target *F. praustnitzii* M21 cells (Fig. 2C).

The specificity of the polyclonal antibody was also evaluated through Western blotting, using total protein extracts obtained according to Sánchez et al. (29), of *Bifidobacterium longum* NCIMB8809, *Collinsella intestinalis* DSM13280, *Escherichia coli* LMG2092, *Blautia coccoides* DSM935, *Lactobacillus acidophilus* DSM20079_T_, *Ruminococcus gauvreauii* DSM19829, *F. prausnitzii* M21, *Bacteroides thetaiotaomicron* VPI-5482, *P. copri* DSM18205, and *Akkermansia muciniphila* DSM26127. Western blot results showed that the polyclonal antibody recognizes the M21 strain, although weak signal background was also detected with some of the other bacteria tested, such as *C. intestinalis* DSM13280, *L. acidophilus* DSM20079_T_, or *B. longum* NCIMB8809 (Fig. S3)

### Detection of *F. praustnitzii* in synthetic microbiotas using flow cytometry.

**FIG 3:**
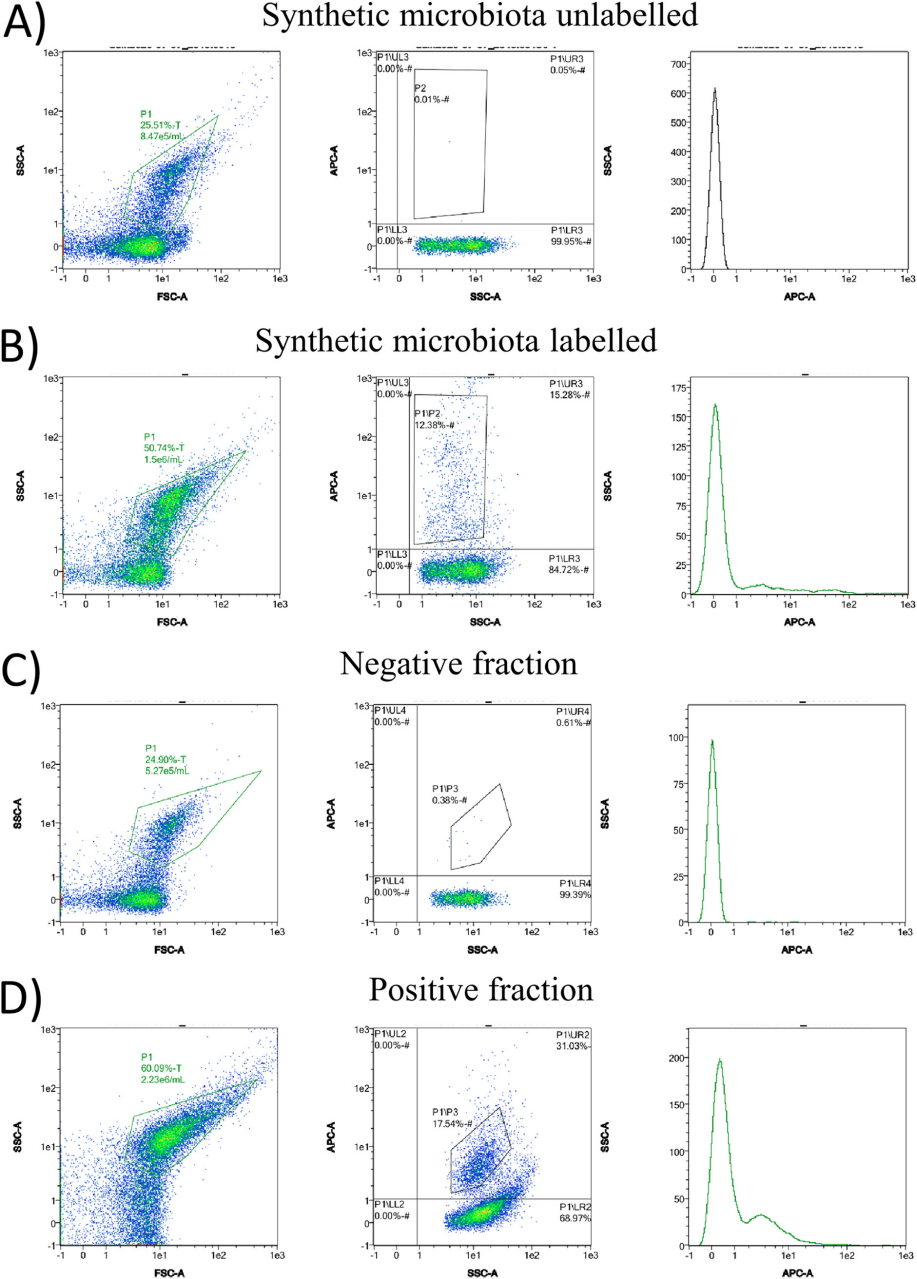
Dispersion diagrams of a representative flow cytometry experiment showing the unlabeled synthetic microbiota (A) and detection of *F. prausnitzii* M21 with the APC-conjugated anti-*F. prausnitzii* M21 antibody in the defined synthetic microbiota (B). Dispersion diagrams of representative flow cytometry experiments showing the APC-specific fluorescence in a synthetic microbiota depleted of *F. prausnitzii* (C) and in the *F. prausnitzii*-enriched fraction (D). On each set of experiments, the following plots are presented: SSC versus FSC (left-most panel), highlighting the P1 gate that identifies events corresponding to bacterial cells; APC-A versus SSC (middle panel), which highlights the P1 events displaying fluorescence signal in the APC channel (P2) and the P1 events not displaying any fluorescence signal (P1/LR3) against the SSC; and number of events versus APC-A (right panel), which represents the number of events identified at each fluorescence intensity in the APC channel. Triplicate experiments were performed.

Given the good specificity of the antibody, we performed labeling of M21 cells when included in a defined bacterial consortium containing representatives of the major human intestinal bacterial phyla: B. longum NCIMB8809, *C. intestinalis* DSM13280 (*Actinobacteria*), E. coli LMG2092 (*Proteobacteria*), *Bl. coccoides* DSM935, L. acidophilus DSM20079^T^, *R. gauvreauii* DSM19829, *F. prausnitzii* M21 (*Firmicutes*), *Bc. thetaiotaomicron* VPI-5482, *P. copri* DSM18205 (*Bacteroidetes*) and *A. muciniphila* DSM26127 (*Verrucomicrobia*). All bacteria were added in an equal 1:1 ratio; therefore, each species represented around 10% of the total bacteria in the mixture. The results show that it is possible to detect *F. prausnitzii* in the synthetic mixture using this antibody, as a percentage of 10.86% ± 1.76% of fluorescently marked events (average of labeling three independent synthetic microbiota mixtures) was obtained in the synthetic microbiota (Fig. 3A and B).

### Enrichment and depletion of *F. prausnitzii* from synthetic microbiota: flow cytometry and microbial profiling analysis.

**FIG 4:**
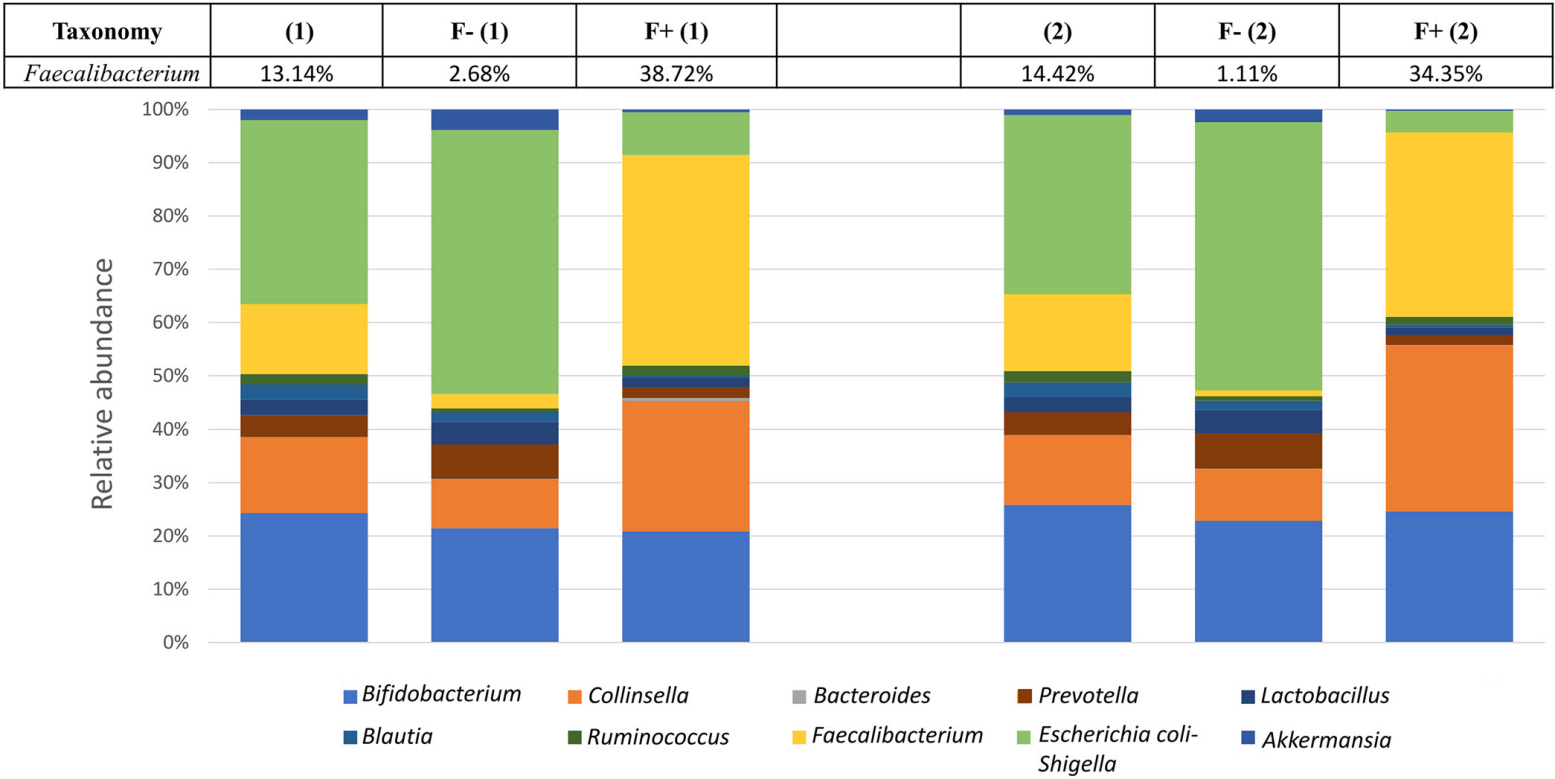
Relative abundance of *F. prausniztii* in two representative samples of synthetic microbiotas and their corresponding depleted (negative) and enriched (positive) fractions.

By combining the use of the anti-*F. prausnitzii* polyclonal antibody conjugated to APC with an anti-APC antibody conjugated to magnetic beads, we were able to deplete the synthetic microbiota from M21 cells in a single step (Fig. 3C). Nevertheless, the positive fraction retained in the magnetic column, in addition to *F. praustnizii* cells, contained other bacteria, such as E. coli and B. longum cells, as evidenced by culture-dependent techniques (data not shown), as shown by detection of events without fluorescence in the APC emission channel in the positive fraction (Fig. 3D). To determine the efficacy of the immunomagnetic capture method herein developed on synthetic microbiotas, the relative abundance of *Faecalibacterium* cells was determined through 16S rRNA sequencing in the original microbiotas and on those modified following immunoseparation of *Faecalibacterium* cells. For this purpose, 16S rRNA sequencing was performed on two independent synthetic microbiota mixtures and on their corresponding negative (*Faecalibacterium*-depleted) and positive (*Faecalibacterium*-enriched) fractions obtained following *Faecalibacterium* immunoseparation. Results from this analysis show that *F. prausnitzii* cells were enriched 2.4- to 3.0-fold in the positive fraction, increasing *Faecalibacterium* representation from 13.78% to 36.53% (average of duplicate experiments), while between 80 and 92% of *F. prausnitzii* cells were depleted in the negative fraction (Fig. 4).

### Enrichment of *F. prausnitzii* from fecal microbiotas as a model to study its contribution to the microbiota immune modulation.

Subsequently, we used our microbiota modification method to establish a new model to investigate the contribution of the *Faecalibacterium* population in a fecal microbiota to its immunomodulatory profile by using PBMCs. For this purpose, we collected fecal microbiotas from healthy donors (HDs) and Crohn’s disease (CD) patients, in which we first analyzed their cell viability (Fig. S4) and microbial profile (Fig. S5). Subpopulations of live and dead cells were not significantly different in both study groups, healthy donors and Crohn’s patients. The percentage of bacteria with an intact membrane in HD microbiotas was 60.54% ± 16.97%, while in CD microbiotas the percentage was 57.28% ± 20.8%. On the other hand, the percentages of cells with active membrane potential were 48.94% ± 18.87% in HD samples and 45.50 ± 18.60 in CD samples. The immunomagnetic separation of *Faecalibacterium* cells from the purified fecal microbiotas coupled to 16S rRNA-based microbial profiling of the *Faecalibacterium*-enriched fractions, allowed us to make a rational selection of four HD microbiotas (with representative enriched relative abundances of *F. prausnitzii*) and six CD microbiotas (with absence of *F. prausnitzii* sequences) for cocultivation with PBMCs. The *F. prausnitzii*-enriched fractions derived from the selected HD microbiotas showed an increase of 2.6 to 4.0 times in the *Faecalibacterium* population (Fig. S5C). Flow cytometry analysis also showed an enrichment of *F. prausnitzii* in the positive fractions, highlighting the possibility of using *F. prausnitzii* polyclonal antibodies for the direct quantification of this bacterium in fecal samples (data not shown).

### Modulation of inflammatory effect of CD microbiota.

**FIG 5:**
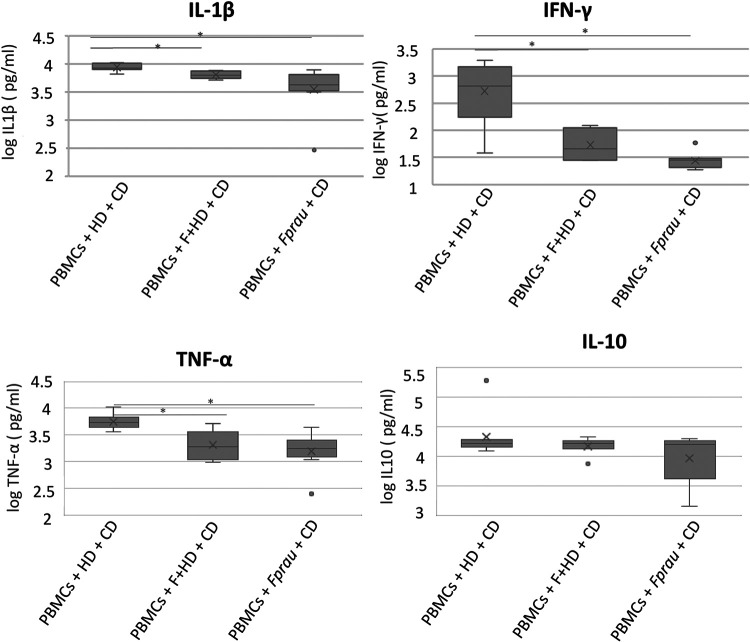
Concentrations of proinflammatory cytokines (IL-1β, IFN-γ, and TNF-α) and the anti-inflammatory cytokine IL-10 of PBMCs cultivated for 24 h with microbiota from healthy donors (HD), microbiota enriched in *F. prausnitzii* (F+HD), or *F. prausnitzii* pure culture (*Fprau*) and subsequently incubated for an additional 24-h period with representative microbiota from Crohn’s disease patients (CD) (see Materials and Methods for detail). Asterisks indicate statistically significant differences between conditions. Experiments were performed with PBMCs from six healthy donors. The differences between the three groups were assessed by *t* test for independent samples.

Available evidence suggests that microbiota from healthy subjects, as well as *F. prausnitzii* cells, might have an immunomodulatory effect, which is hypothesized to counteract the inflammatory response induced by the microbiota from CD patients. To evaluate this possible regulation, the effect of preincubating PBMCs with different microbiotas and *F. prausnitzii* prior to exposing them to CD microbiota was also analyzed. To do this, a sample representative of a healthy microbiota (obtained from pooling 4 HD samples), a *Faecalibacterium-*enriched microbiota (a pool of the 4 positive fractions obtained after the immunoseparation-based *Faecalibacterium* enrichment step from the selected HD microbiotas), or an *F. prausnitzii* pure culture was added to PBMCs. Subsequently, after 24 h of prestimulation with these HD or *Faecalibacterium* cultures, a microbiota representative of CD microbiotas (obtained from pooling 6 CD samples; see Materials and Methods for detail) was added to the system for an additional 24-h incubation period. Afterwards, a panel of 9 cytokines was evaluated in cell culture supernatants in order to determine the differential immunostimulatory capacity of CD microbiota following preincubation with different variations of healthy microbiotas. The results showed that PBMC preincubations with the healthy microbiota enriched in *Faecalibacterium* and with a pure culture of *F. prausnitzii* are able to significantly reduce the proinflammatory effect induced by CD microbiota, as these conditions showed lower levels of the proinflammatory cytokines interleukin-1β (IL-1β), gamma interferon (IFN-γ), and tumor necrosis factor alpha (TNF-α) than the effect produced by preincubating the PBMCs exclusively with the microbiota of healthy donors (Fig. 5). In the case of anti-inflammatory cytokine IL-10, no significant differences were observed among conditions. Differences were not observed with the rest of the cytokines (IL-2, IL-4, IL-6, IL-12p70, and IL-17A [CTLA-8]) (data not shown).

## DISCUSSION

In this study, a methodology developed in our group to enrich or deplete a microbiota in a given taxon (30) was used to explore *in vitro* the immunomodulatory role of *Faecalibacterium* within the gut microbiota. The reduction of butyrate-producing bacteria, including some representative *Faecalibacterium* species in particular, has been associated with the presence of chronic diseases, including depression, obesity, type 2 diabetes, and IBD (12, 31–33). Some *Faecalibacterium* species, including *F. prausnitzii*, are considered among the most important bacterial indicators of a healthy gut. The beneficial effects of these species include their ability to produce butyrate, which favorably modulates the intestinal immune system, oxidative stress, and colonocyte metabolism (34, 35). Besides, over the last few years an increasing number of studies have prompted interest in considering this bacterium as a next-generation probiotic (9, 14, 36). Given the relevance of this bacterium, we chose a representative strain of the species *F. prausnitzii* as a model microorganism to demonstrate that we can selectively modify the microbial content of the fecal microbiota and use this targeted modification to establish a new model to study microbe-host interactions.

The results show that our methodology allows enrichment of the *Faecalibacterium* population in real fecal microbiotas, although it did not allow complete depletion *Faecalibacterium* cells from the sample, which was not even achieved after multiple rounds of enrichment with this methodology (data not shown). This may be due to a range of different facts. On the one hand, there may exist important heterogeneity in the population of *Faecalibacterium* cells, what can be particularly problematic when working with natural fecal microbiotas. This heterogeneity could mean that not all *Faecalibacterium* cells within a sample have the same phenotype, and therefore they can be recognized by the antibody in a different manner (37). It is also worth remarking that although by the time this investigation was conducted only one species, *F. prausnitzii*, was recognized within the genus *Faecalibacterium*, to date at least six different species have been ascribed to this genus. Since these species present relatively high degrees of homology and considering that the anti-*Faecalibacterium* sera was generated against whole-cell envelopes of a given *Faecalibacterium* strain, we would expect this serum not to be species specific. However, we cannot rule out the possibility that some *Faecalibacterium* species might not be recognized as efficiently as the M21 strain. Assessment of the validity of our approach and antibody with other representative strains/species of *Faecalibacterium* will aid in determining the whole range of specificity of the antibody used and to establish whether the whole *Faecalibacterium* population or only some of the species are being recognized and captured by this antibody.

Uncomplete depletion of the *Faecalibacterium* population could also be consequence of the fact that not all cells are accessible to the antibody due to aggregation phenomena. In this regard, immunomagnetic capture does not allow the isolation of this bacterium alone, since the *F. prausnitzii-*enriched fraction is coeluted with a background microbiota composed of some of the bacterial members of the original microbiota. This could be due to the fact that the antibody is not completely specific against *F. prausnitzii* or to cell-to-cell interaction phenomena that provoke stable associations between microorganisms. These coaggregation phenomena may lead to the coisolation of *Faecalibacterium* cells with additional species in the positive fractions, but coaggregation phenomena may also mask the *F. prausnitzii* antigens recognized by the antibody used in this work, preventing its efficient capture from the microbiota sample. This limitation of our technology has been previously shown with fecal microbiota samples enriched in Lactobacillus acidophilus and needs further refinement to minimize coelution with other taxa. Although the coelution can be avoided by trypsinization, this treatment compromises the viability of the bacterial cells and therefore could have an influence on downstream experiments (30).

Knowing that our method can be used to specifically modify the relative abundance of a target taxon, we wanted to check if it allows establishment of a new model to study, *in vitro*, the contribution of a specific gut bacterium to a given biological effect. Sokol and coworkers evaluated the immunomodulatory properties of *F. prausnitzii in vitro* (cellular models) and *in vivo* (mice with 2,4,6-trinitrobenzenesulfonic acid-induced colitis). *In vitro*, their results showed that PBMC stimulation by Faecalibacterium duncaniae A2-165 led to significantly lower IL-12 and IFN-γ production levels and higher secretion of IL-10 (11). Qiu and colleagues evaluated the anti-inflammatory and immunomodulatory capacities of *F. prausnitzii* by comparing them with those of B. longum *in vitro* (PBMC models) and *in vivo* (rat model of 2,4,6-trinitrobenzenesulfonic acid-induced colitis). Their results showed that *F. prausnitzii*, B. longum, and *F. prausnitzii* supernatant facilitated the induction of IL-10 and TGF-β1, while induced relatively mild production of IL-12p70 in both cellular and animal models (38). In line with these findings, cells or cell-free supernatants of several *Faecalibacterium* species and strains have been reported to reduce the severity of acute (11), chronic (24), and low-grade (26) chemical-induced inflammation in murine models. Taken advantage of the possibilities of our methodology, we also set up a cell model to test the immunomodulatory capacity of the modified microbiotas in coculture with PBMCs. PBMCs only partially represent the population of immunocompetent cells at the intestinal environment; however, they are easy, affordable, and convenient models to approach the investigation of the gut mucosal immune response to bacteria (39, 40). To do that, we incubated PBMCs in the presence of different microbiotas (to adapt the immune cells to the presence of gut microbes) and subsequently challenged the cells with CD microbiota, which has been demonstrated to display a proinflammatory behavior in different models (41, 42). We observed that preincubation of PBMCs with the *F. prausnitzii*-enriched microbiota, as well as with the *F. prausnitzii* pure culture, exerted an effect on the PBMCs that led them to display an anti-inflammatory phenotype, with a concomitant reduction of inflammatory cytokines, following a subsequent exposure to proinflammatory CD microbiota. These results support the feasibility of our approach to study bacterium-host interactions. It is worth highlighting that we did not use the *F. prausnitzii*-depleted fractions in our experimental setup because they still contain a small population of *F. prausnitzii* cells, but the use of the depleted population could be a reasonable option to study the effect of the lack of a specific microorganism in a complex microbial consortium. On the other hand, the high levels of IL-10 in PBMCs supplemented with microbiotas of patients with CD and with those from healthy donors are in accordance with the results of other authors. For example, Wang and colleagues found that CD patients had significantly higher levels of IL-10 compared to controls (43) and Kucharzik and coworkers reported increased serum IL-10 concentration in patients with active CD or ulcerative colitis (UC) compared to controls (44). The reason for the increase in these cytokines is unclear. However, other authors did not find elevated levels of IL-10 in patients with CD. For example, Nielsen et al. reported that serum IL-10 concentrations did not differ among UC or CD patients and healthy controls (45). The discordance between studies could be due to differences in the age of the donors, in the severity of the disease, or in the phase of disease, among others.

Our results with targeted modified intestinal microbiotas enriched in *Faecalibacterium* cells support that this bacterial group exerts anti-inflammatory effects on its own, but it can also contribute to the anti-inflammatory effect of a complex microbiota under the conditions used in this study. These results are promising as they confirm that the immunoseparation approach developed herein, in combination with appropriate models, allows the study of the biological effect of a given species on a given disease, such as IBD. This also opens new therapeutic possibilities linked to the targeted modification of the intestinal microbiota. Independently of the potential that our methodology can hold to deplete or enrich a specific bacterial subpopulation in complex microbial communities, it is worth highlighting that it can provide a number of additional advantages over existing techniques. First, it is an easy and affordable method that does not need high-cost equipment or facilities as, once properly optimized and validated, the microbiota fractionations rely on a magnetic immunoseparation step performed on simple portable devices that can be manipulated within the anaerobic hood, preventing the necessity of conducting flow cytometry assays on the obtained fractions. Therefore, we postulate that such methodologies could be implemented in standard microbiology laboratories. This is a remarkable advantage compared with methods using fluorescence-activated cell sorting, some of which have already been reported in the literature (46). Second, our method allows the study of host-microbiota interactions from a completely new perspective: by depleting/enriching specific populations in the microbiota, we can infer their role within the community. Also, it is a transversal methodology that can be applied to any microbial community composed of culturable microorganisms, fastidious, or even nonculturable members if reverse-genomics procedures are applied (47). Further refinement of the procedure herein developed may be required so as to ensure preservation of the viability and metabolic activity of particularly fastidious taxa, such as EOS species, to ensure maintenance of their contribution to gut microbiota functionalities in downstream models of activity. To the best of our knowledge, this is the first work showing that immunomagnetic capture of gut commensal bacteria allows a targeted enrichment/depletion from fecal microbiota samples, establishing a new model to explore cross talk interactions between bacteria and eukaryotic cells.

## MATERIALS AND METHODS

### Ethics statement.

Ethics approval for this study (reference code AGL2016-78311-R) was obtained from the Bioethics Committee of CSIC (Consejo Superior de Investigaciones Científicas) and from the Regional Ethics Committee for Clinical Research (Servicio de Salud del Principado de Asturias, no. 127/16) in compliance with the Declaration of Helsinki. All determinations were performed with fully informed written consent from all participants involved in the study.

### Bacterial strains and growth conditions.

Lactobacillus acidophilus DSM20079^T^ was grown in MRS (Biokar Diagnostics, France). Bifidobacterium longum biotype *longum* NCIMB8809 was grown in MRS supplemented with 0.05% (wt/vol) l-cysteine (MRSC) (Sigma, St. Louis, MO). Blautia coccoides DSM935 and Bacteroides thetaiotaomicron DSM2079 were cultivated in a combination of Oxoid reinforced clostridial broth (Oxoid, Ltd., Basingstoke, Hampshire, United Kingdom) and brain-heart infusion medium (BHI) (Oxoid, Ltd., Basingstoke, Hampshire, United Kingdom) supplemented with 5% (vol/vol) heat-inactivated fetal bovine serum (FBS) (LabClinics, Barcelona, Spain). Akkermansia muciniphila DSM26127 was grown in Oxoid anaerobe basal broth (Oxoid, Ltd., Basingstoke, Hampshire, United Kingdom). Faecalibacterium prausnitzii M21 was growth in BHI medium supplemented with 0.5% (wt/vol) yeast extract, 1 g/L cellobiose (PanReac AppliChem, Darmstadt, Germany), 1 g/L maltose (PanReac AppliChem, Darmstadt, Germany), and 0.5 g/L cysteine. Collinsella intestinalis DSM13280, Prevotella copri DSM18205, and Ruminococcus gauvreauii DSM19829 were growth in Gifu anaerobic medium (GAM) (Nissui Pharmaceutical Co, Tokyo, Japan) supplemented with 0.05% (wt/vol) l-cysteine (Sigma, St. Louis, MO). All of these bacteria were first grown on the surface of agar plates from −80°C stocks in an MG500 anaerobic chamber (Don Whitley Scientific, West Yorkshire, United Kingdom) in an atmosphere of 10% (vol/vol) H_2_, 10% CO_2_, and 80% N_2_ for 48 h at 37°C. These precultures were used as fresh inoculum for the preparation of active cultures. Escherichia coli LMG2092 was grown in Luria-Bertani broth (LB) containing 10 g/L tryptone (Biokar Diagnostics, France), 5 g/L yeast extract (Biokar Diagnostics, France), and 10 g/L of NaCl (Merck, KGaA, Darmstadt, Germany), with constant shaking of 200 rpm for 24 h at 37°C.

For the flow cytometry experiments, bacteria were harvested by centrifugation at the early or mid-exponential growth phase, corresponding to an optical density at 600 nm (OD_600_) of 0.7 (Fig. S1). Pellets were washed twice in bacterial flow cytometry buffer (Miltenyi, Bergisch Gladbach, Germany) and resuspended in the same buffer to an adjusted OD_600_ of 0.2, which represents around 1E10^8^ CFU/mL.

### *F. prausnitzii* cell surface preparation.

Cell surfaces of *F. prausnitzii* M21 were obtained using a mixture of chloroform and methanol through an adapted method of Wessel and Flügge (48). Fifty-milliliter aliquots of fresh BHI medium supplemented with 0.5% (wt/vol) yeast extract, 1 g/L cellobiose, 1 g/L maltose, and 0.5 g/L cysteine were inoculated (1% [vol/vol]) from an overnight culture. Cultures were allowed to enter the stationary phase of growth (24 h), and cells were harvested by centrifugation (3,500 rpm, 4°C, 10 min). Pellets were resuspended in 1 mL of chloroform, 4 mL of methanol, and 3 mL of water, and the resultant cell suspensions were maintained in a rotator disk for 2 h at room temperature. Then, cells were collected by centrifugation at 3,500 rpm for 5 min. The pellet was transferred to one microcentrifuge tube, washed with phosphate-buffered saline (PBS) at 13,000 rpm for 5 min, and finally resuspended in PBS.

### Polyclonal antibody generation.

A polyclonal serum against the cell surface of *F. prausnitzii* M21 was generated at the Central Facilities of the University of Oviedo (Spain). A rabbit was immunized five times, with an interval of 15 days between immunizations, with 500 μg of M21 cell surface preparation dissolved in 1 mL of PBS and mixed with 1 mL of Freund's incomplete adjuvant. Polyclonal serum was obtained as previously described (30). Briefly, the rabbit was sacrificed by intracardiac puncture and blood was let to coagulate. Serum was separated by centrifugation and used for purification of the IgG fraction. First, ammonium sulfate was added to a final concentration of 45% (wt/vol), and the mixture was incubated overnight at 4°C. After centrifugation, the pellet was resuspended in PBS, extensively dialyzed, and purified through a protein A Sepharose 4 Fast Flow system and a Vivaspin 20 device (molecular weight cutoff of 10 kDa). The protein concentration was estimated by measuring the *A*_280_ of the sample in a Shimadzu spectrophotometer (UV-1280 model), and serum was aliquoted and stored at −80°C.

### Antibody labeling.

The IgG fraction of the polyclonal serum against the *F. prausnitzii* M21 strain was conjugated with allophycocyanin (APC) using an APC conjugation kit (Abcam, Cambridge, MA, USA) following the manufacturer’s instructions. APC has an absorbance maximum at 650 nm and a fluorescence emission peak at 660 nm. For APC conjugation, the polyclonal antibody was reconstituted in amine-free phosphate-buffered saline (PBS) at 1.5 mg/mL. Then, 10 μL of APC-modifier reagent was added to 100 μL of the antibody suspension, and this suspension was directly added to the reactive dye for conjugation. The antibody-dye mixtures were incubated in the dark at room temperature (20 to 25°C) for 3 h. After incubation, 10 μL of APC-quencher reagent was added and mixed gently.

### Bacterial labeling.

One milliliter of cultures in exponential phase of growth (OD_600_ of ~0.7) was centrifuged at 10,000 × *g* for 5 min. After that, bacterial suspensions were adjusted to an OD_600_ of 0.2 (around 1.10^8^ CFU/mL) using PBS. Twenty-five microliters of bacterial suspension was mixed with 25 μL of the APC-conjugated antibody at a final concentration of 20 μg/mL for flow cytometry. The samples were incubated for 15 min at room temperature and then were washed with PBS. Finally, bacteria were resuspended in 150 μL of PBS for flow cytometry analyses.

### Flow cytometry analysis.

Bacterial cells labeled with anti-*F. prausnitzii* M21 polyclonal antibody were acquired and analyzed in a MACS Quant flow cytometer device (Miltenyi Biotec, Germany) using the following acquisition parameters: flow rate set to low, uptake volume of 10 μL, FSC set to hyperlogarithmic amplification (370 V), SSC set to hyperlogarithmic amplification (440 V), and channel R1 corresponding to the APC detection set (filtering wavelengths from 655 to 730 nm) to hyperlogarithmic amplification (350 V).

### Immunofluorescence microscopy.

The binding specificity of the antibodies contained in the polyclonal serum was also determined with a confocal scanning laser microscope equipped with a Leica DFC365FX digital camera (DMi8; Leica Microsystems). First, *F. prausnitzii* M21 was grown until it reached an exponential-phase OD_600_ of ~0.7, and then cells were collected, washed with PBS, and incubated for 15 min with the polyclonal antibody conjugated with APC (200 μg/mL). The cells were washed and resuspended in 10 μL of PBS and analyzed with fluorescence microscopy using a 100× oil objective. The images were acquired with software LasX (Leica Microsystems). The APC filter cube (excitation, 620/60; emission, 700/75) was used for labeled *F. prausnitzii.*

### Specificity of polyclonal antibody by Western blotting.

Sodium dodecyl sulfate-polyacrylamide gel electrophoresis (SDS-PAGE) and Western blotting were performed as described below. Total proteins were obtained following a previously described protocol (29). Proteins were separated using a polyacrylamide concentration of 12.5% (wt/vol) (Invitrogen precast gels; Thermo Fisher Scientific, Waltham, MA, USA) for 1 h at a constant intensity of 40 mA/gel. Proteins were visualized with Coomassie blue R-250 (Thermo Fisher Scientific, USA). Proteins separated by SDS-PAGE were transferred and immobilized onto polyvinylidene fluoride (PVDF) membranes (GE Healthcare, Madrid, Spain) and hybridized following previously described methods (49), using a dilution of 1:2,000 of the polyclonal antibody and developed with a secondary antibody (horseradish peroxidase-conjugated anti-rabbit or anti-mouse IgG from Sigma-Aldrich) and a commercial solution containing the chromogenic reagents chloronaphthol and diaminobenzidine (CN/DAB substrate kit; Thermo Fisher Scientific).

### Fecal sample collection and microbiota extraction.

Ten fecal samples from healthy donors (HDs) and 10 fecal samples from CD patients with active disease were used in this study. Healthy donors did not report any severe disease and had not consumed antibiotics in the past 6 months. Fresh fecal material was collected in a sterile container and immediately manipulated and homogenized within a maximum time span of 2 h from collection. Nine milliliters of sterile NaCl (0.9% [wt/vol]) was added to 1 g of sample, and the mixture was homogenized in a sterile bag, using a laboratory paddle blender (Stomacher Lab Blender 400, Seward, Ltd., United Kingdom) for 1 min. Microbiota extraction was then performed following the protocol described by Hevia and coworkers, which was previously demonstrated not to significantly alter the viability of the extracted microbiotas (50). Briefly, a solution of 80% (wt/vol) Nycodenz (PROGEN Biotechnik GmbH, Heidelberg, Germany) was prepared in ultrapure water and sterilized at 121°C for 15 min. A volume of 3 mL of the diluted, homogenized fecal sample was placed on top of 1 mL of the Nycodenz solution, and the mixture was centrifuged for 40 min at 4°C at 10,000 × *g* in an MLS-50 swinging-bucket rotor (Beckman Coulter, Indianapolis, IN, USA). The upper phase (soluble debris) was discarded after centrifugation, and the layer corresponding to the microbiota was collected, washed once, and resuspended in 1 mL of PBS.

### Viability of the fecal microbiota in CD versus healthy conditions.

The viabilities of the microbiotas from 10 HD and 10 CD patients were analyzed by flow cytometry after density gradient extraction (50). Microbiotas were labeled using two kits to differentiate cells with noncompromised membrane integrity/compromised membranes and metabolically active/inactive bacteria: the LIVE/DEAD BacLight bacterial viability kit and the BacLight bacterial membrane potential kit (Thermo Fisher Scientific, Waltham, MA, USA). The LIVE/DEAD BacLight bacterial viability kit was used to stain live bacteria with SYTO9 and dead bacteria with propidium iodide (PI). The BacLight bacterial membrane potential kit provides DiOC2(3) (3,3′-diethylosa-carbocynine iodide), which stains metabolically active bacteria with red and green fluorescence, while inactive bacteria stain with only green fluorescence. Labeled microbiotas were acquired (10,000 events per sample) and analyzed in a MACS Quant flow cytometer device (Miltenyi Biotec, Germany) using the following acquisition parameters: flow rate set to low, uptake volume of 10 μL, FSC set to hyperlogarithmic amplification (370 V), SSC set to hyperlogarithmic amplification (440 V), channel B1 corresponding to fluorescein isothiocyanate (FITC) (342 V), and channel B3 corresponding to the phycoerythrin (PE)-Vio 615 (360 V) for the analysis of noncompromised/compromised membrane of bacteria and FITC (370 V) and B3 (480 V) for the analysis of metabolically active/inactive bacteria.

### Specific enrichment and depletion of *F. prausnitzii* from synthetic microbiota and fecal microbiota.

For the enrichment and depletion of *F. prausnitzii* from synthetic microbiotas, a mixture of 10 bacteria, including representative species of the main phyla encountered in the healthy human gut microbiota (51) was formulated as a synthetic microbiota. Specifically, this mixture included L. acidophilus DSM20079^T^ (phylum *Firmicutes*), B. longum NCIMB8809 (phylum *Actinobacteria*), *Bl. coccoides* DSM935 (phylum *Firmicutes*), *Bc. thetaiotaomicron* DMS2079 (phylum *Bacteroidetes*), *A. muciniphila* DSM26127 (phylum *Verrucomicrobia*), *F. prausnitzii* M21 (phylum *Firmicutes*), *C. intestinalis* DSM13280 (phylum *Actinobacteria*), *P. copri* DSM18205 (phylum *Bacteroidetes*), *R. gauvreauii* DSM19829 (phylum *Firmicutes*), and E. coli LMG2092 (phylum *Proteobacteria*). For preparation of the synthetic microbiota mixture, actively growing cells from each bacterium species were collected at an early to mid-exponential phase (OD_600_ ≈ 0.7), bacterial suspensions were adjusted to an OD_600_ of 0.2, and then these suspensions were mixed in a volumetric 1:1 ratio (10% of each bacterium). Then, these mixtures were labeled with 1 μL of polyclonal anti-*F. prausnitzii* serum IgG fraction conjugated to APC and incubated for 15 min at room temperature. To test the reproducibility of the labeling, at least three independent replicate analyses were carried out. For the enrichment and depletion of *F. prausnitzii* from human fecal microbiotas, cell suspensions extracted as previously described were adjusted to an OD_600_ of 5.0. *F. prausnitzii*-enriched fractions and *F. prausnitzii*-depleted fractions were designed as positive and negative fractions, respectively. Then, labeling was performed with 10 μL of the polyclonal anti-*F. prausnitzii* serum IgG fraction conjugated to APC and incubated for 15 min at room temperature. In both cases of synthetic and fecal microbiotas, mixtures (labeled samples) were washed at 13,000 × *g* for 5 min and the supernatants removed. For positive selection (enrichment) of *F. prausnitzii*, labeled microbiotas were resuspended in 500 μL of PBS. To this mixture, 250 μL of magnetic anti-APC particles was added (BD Biosciences, San José, CA, USA). The mixture was incubated for 15 min at 20°C. Then, bacteria were collected and washed at 13,000 × *g* for 5 min with PBS and finally resuspended in 500 μL of PBS. For the immunomagnetic separation of *F. prausnitzii* from this suspension, magnetic separation (MS) columns, commonly used for the positive selection of magnetically labeled cells, were placed on a MiniMACS separator (Miltenyi) and conditioned with 500 μL PBS prior to addition of the labeled cell suspensions. The positive fraction, containing magnetic anti-APC particle-labeled cells, was retained in the magnetic columns, while the negative fraction washed through the MS column. The column was washed three times with PBS, and then the positive fraction was eluted by separating the magnetic columns from the MiniMACS separator and adding 500 μL of PBS. Both positive and negative fractions were analyzed by flow cytometry and metataxonomics.

The purified microbiotas and the negative (*Faecalibacterium*-depleted) and positive (*Faecalibacterium*-enriched) fractions collected during the immunoseparation procedure were washed twice in PBS and stored in the same buffer in aliquots of 1 mL of 1E08 CFU/mL at −80°C. Bacterial cells on each fraction/sample were counted by using a Neubauer improved cell counting chamber (Marienfeld Superior, Germany). For PBMC assays, bacteria from fecal microbiota samples were inactivated prior to adding them to PBMCs in order to prevent bacterial overgrowth in the PBMC cultures, as this could affect the PBMCs’ viability and responses. For this purpose, the fecal microbiota was inactivated by exposure to three consecutive cycles of 30 min under radiation in a UV chamber (15W; Selecta, Barcelona, Spain) as previously described (39). Plate counting was carried out after UV treatment to corroborate the absence of bacteria able to recover in proper medium, which would suggest that most bacterial cells in the samples were incapable of sustaining growth under laboratory conditions and thus are not likely to interfere with the PBMCs’ activity in the cocultivation models. UV-killed bacterial suspensions were distributed in aliquots and stored at −80°C until use.

### DNA extraction and 16S rRNA gene partial sequencing from synthetic and fecal microbiotas, and from positive and negative fractions.

DNA was isolated from each of the synthetic microbiotas and from their corresponding positive and negative fractions using a method based on the GenElute bacterial genomics DNA kit (Sigma, St. Louis, MO). The samples were resuspended in 200 μL of lysozyme solution (45 mg/mL) and incubated at 37°C for 30 min. Twenty microliters of proteinase K (20 mg mL^−1^) and 200 μL of lysis solution C were added, and the mixture was incubated at 55°C for 10 min. DNA was then purified using nucleic acid binding columns following the manufacturer’s instructions. For fecal microbiotas and their positive and negative fractions, DNA was extracted with the Fast DNA stool minikit (Qiagen, Valencia, CA, USA) following the manufacturer’s protocols, with a minor modification: after the addition of proteinase K and buffer AL, the samples were incubated at 70°C for 30 min, and subsequently, ethanol was added to the lysate and the lysate was placed carefully into the QIAamp spin column.

For synthetic microbiota, partial 16S rRNA gene sequences were amplified from extracted DNA using primer pair Probio_Uni/Probio_Rev, targeting the V3 region of the 16S rRNA gene sequence. The use of these primers was previously optimized using single strains and primer matching analysis for the different taxa composing the synthetic microbiota (52). For fecal microbiota, partial 16S rRNA gene sequences were amplified from extracted DNA using a primer pair targeting the V3-V4 region of the 16S rRNA gene sequence: 16S-ProV3V4-Fw (CCTACGGGNBGCASCAG) and 16S-ProV3V4-Rev (GACTACNVGGGTATCTAATCC) (53). For all sample types, 16S rRNA gene sequencing was performed using Illumina MiSeq equipment according to previously reported protocols (49). Following sequencing, the fastq files were processed using a custom script based on the QIIME software suite2 (54). Briefly, paired-end read pairs were assembled. Then, a quality control filtering step was applied to retain sequences with a length between 140 and 400 bp and a mean sequence quality score of >20, while sequences with homopolymers of >7 bp and mismatched primers were omitted. In order to calculate downstream diversity measures (α- and β-diversity indices), 16S rRNA amplicon sequence variants (ASVs) were defined at 100% sequence homology using DADA2 (55) and ASVs not encompassing at least 2 sequences in the same sample were removed. All reads were classified to the lowest possible taxonomic rank using QIIME2 (54, 56) and a reference data set from the SILVA database v.132 (57). The raw sequences generated have been deposited in the NCBI Short Read Archives repository.

### Peripheral blood mononuclear cell isolation.

An *in vitro* model with PBMCs was used to study how the modification of *Faecalibacterium* representation in fecal microbiotas affected their interaction with the host immune system. For this purpose, PBMCs were isolated from the buffy coat of 6 healthy donors obtained from the Community Center for Blood and Tissues of Asturias (Oviedo, Spain). Donors classified as healthy are individuals aged between 18 and 65 years old with absence of diseases incompatible with blood donation and without any pharmacological treatment. For this, 5.0 mL of buffy coat was diluted in 5 mL of PBS, and 10 mL of homogenate was deposited on top of 5 mL of Ficoll-Hypaque Plus (Nycomed, Oslo, Norway) for gradient separation. PBMCs were isolated by centrifugation at 1,800 rpm for 30 min on Ficoll-Hypaque Plus with two further steps of washing with PBS: the first at 1,200 rpm for 10 min to discard platelets and the second at 1,500 rpm for 5 min. Then, PBMCs were counted in the Neubauer chamber and resuspended in RPMI 1640 supplemented with 10% FBS (Biowest, Rue de la Caille, France) and antibiotics (penicillin, streptomycin, amphotericin B; HyClone, GE Healthcare Europe GmbH, Barcelona, Spain) at a final concentration of 2E06 cells/mL.

### Cocultivation of PBMCs and microbiotas.

PBMCs from six donors were used for this analysis. PBMCs from each donor were cultivated in flat-bottom 96-well microplates using a 100-μL cell suspension (2E06 cells/mL per well). Positive (LPS 1.0 μg/mL) and negative (no stimulus) controls were included for each donor. Three different pools of purified microbiotas were produced: (i) the pool of microbiotas from healthy donors (HDs) was generated by mixing an equivalent number of microbial cells from the microbiotas HD2, HD3, HD4, and HD5 (corresponding to those samples for which the highest enrichment of *Faecalibacterium* representation in the positive fraction was achieved), (ii) the pool of *F. prausnitzii*-enriched fractions (F+HD) was generated with the positive fractions of the four previously mentioned HD microbiotas, and (iii) the pool of microbiotas from Crohn’s disease (CD) patients was generated by mixing equivalent numbers of microbial cells from the microbiotas CD1, CD2, CD3, CD8, CD9, and CD10 (corresponding to those samples where *Faecalibacterium* could not be detected). Quantification of the cell contents in the different microbiotas was carried out with a Neubauer improved cell counting chamber. The pooled microbiotas were inactivated by exposure to three consecutive cycles of 30 min under radiation in a UV chamber (15W; Selecta, Barcelona, Spain), according to López et al. (39). Subsequently, 5 μL of inactivated bacterial suspensions (1E08 bacterial cells/mL) was added to PBMCs, to reach a ratio of mononuclear cells to bacterial cells of 1:2.5. The microplates were incubated at 37°C with 5.0% CO_2_. After 24 h of incubation of PBMCs in the presence of HD microbiota, F+HD microbiota, or pure culture of *F. prausnitzii*, the CD microbiota was added and then samples were incubated for additional 24 h. At the end of the incubation period, cytokines were measured as described below.

### Cytokine determination.

Cytokine determination was carried out with a Luminex system using the 9-plex magnetic Procarta immunoassay kit (Affymetrix). All samples were acquired and analyzed using a Luminex 200 (Luminex Corp., Austin, TX, USA). The quantification of IFN-γ, IL-1β, IL-2, IL-4, IL-6, IL-10, IL-12p70, IL-17A (CTLA-8), and TNF-α was carried out following the manufacturer’s instructions.

### Statistical analysis.

Statistical analysis of the cytokine data was performed using IBM-SPSS version 25.0 (SPSS, Inc., Chicago). The Shapiro-Wilk test was used to assess the normal distribution of the data, evidencing a nonnormal distribution for all tested variables (*P* < 0.05). Accordingly, a nonparametric Mann-Whitney U test was used to determine differences among groups (PBMCs+HD+CD, PBMCs+F+HD+CD, and PBMCs+Fprau+CD). Significance was declared at a *P* value of <0.05 for all analyses.

### Data availability.

Raw sequences generated in this study have been deposited in the NCBI Short Read Archives repository under BioProject accession no. PRJNA891498.
