# Supplementary material for: Immunomagnetic Capture of Faecalibacterium prausnitzii Selectively Modifies the Fecal Microbiota and Its Immunomodulatory Profile
Source: Microbiol Spectr. 2023 Jan 4;11(1):e01817-22. doi: 10.1128/spectrum.01817-22 (PMC9927134; doi:10.1128/spectrum.01817-22)
Supplement: Supplemental file 1 — Supplemental material. Download spectrum.01817-22-s0001.pdf, PDF file, 4.9 MB [file spectrum.01817-22-s0001.pdf]

Immunomagnetic capture of *Faecalibacterium prausnitzii* selectively modifies the fecal microbiota and its immunomodulatory profile

Raquel Marcos-Fernández[1,2], Sabino Riestra[2,3], Rebeca Alonso-Arias[4,5*], Lorena Ruiz[1,2,*], Borja Sánchez[1,2], Abelardo Margolles[1,2]

1. Departamento de Microbiología y Bioquímica, Instituto de Productos Lácteos de Asturias, Consejo Superior de Investigaciones Científicas (IPLA-CSIC), Paseo Río Linares s/n 33300, Villaviciosa, Asturias, Spain.

2. Instituto de Investigación Sanitaria del Principado de Asturias (ISPA), Avenida Hospital Universitario s/n 33011, Oviedo, Asturias, Spain.

3. Departamento de Gastroenterología, Unidad de Enfermedad Inflamatoria Intestinal, Hospital Universitario Central de Asturias (HUCA), 33011, Oviedo, Asturias, Spain.

4. Departamento de Inmunología, Hospital Universitario Central de Asturias (HUCA), 33006, Oviedo, Asturias, Spain.

5. Department of Cardiac Pathology, Health Research Institute of the Principality of Asturias (ISPA), Oviedo, Asturias, Spain

* Corresponding authors: ralonsoarias@hotmail.com; lorena.ruiz@ipla.csic.es

Supplementary figure 1. Growth curves of the bacterial species used to build a synthetic microbiota. Each species/strain was inoculated from freshly overnight grown pre-cultures, in its corresponding media as indicated in the material and methods section. Cultures were incubated at 37ºC in an anaerobic chamber (Don Whitley Scientific, West Yorkshire 100, UK; atmosphere of 10% (v/v) H_2, 10% CO_2, and 80% N_2) and their growth was monitored during 80 hours by measuring OD at 600 nm at regular intervals by using a turbidimeter installed within the anaerobic incubator. Growth curves present data of at least independent biological triplicates.

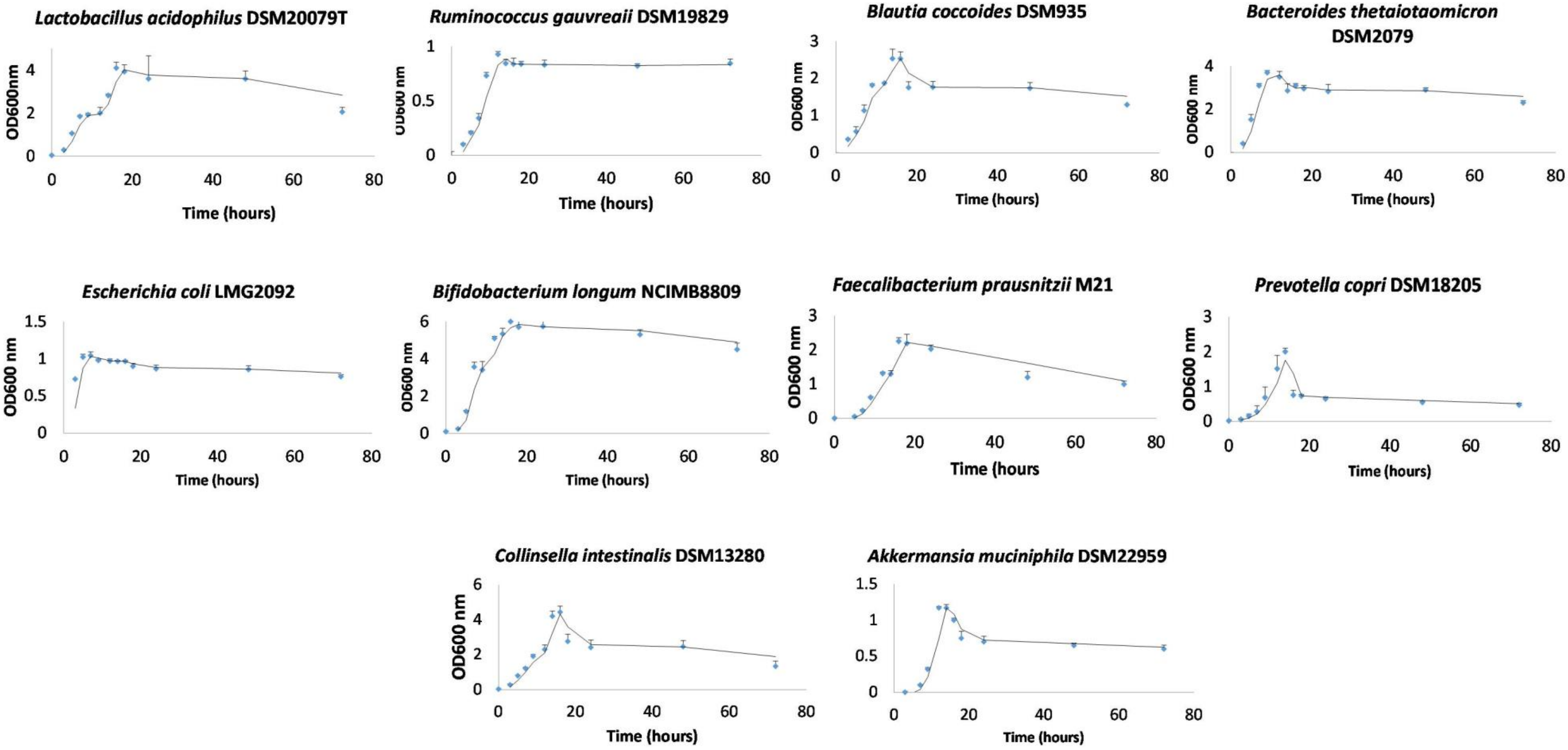
Lactobacillus acidophilus DSM20079T
OD600nm
Time (hours)
Ruminococcus gauvreaii DSM19829
OD600 nm
Time (hours)
Blautia coccoides DSM935
OD600 nm
Time (hours)
Bacteroides thetaiotaomicron DSM2079
OD600 nm
Time (hours)
Escherichia coli LMG2092
OD600 nm
Time (hours)
Bifidobacterium longum NCIMB8809
OD600 nm
Time (hours)
Faecalibacterium prausnitzii M21
OD600 nm
Time (hours
Prevotella copri DSM18205
OD600 nm
Time (hours)
Collinsella intestinalis DSM13280
OD600 nm
Time (hours)
Akkermansia muciniphila DSM22959
OD600 nm
Time (hours)

Supplementary figure 2. Dispersion diagrams of representative flow cytometry experiments showing the acquisition of labelled and non-labelled *L. acidophilus* (A), *B. longum* NCIMB8809 (B), *A. muciniphila* DSM26127 (C), *C. intestinalis* DSM13280 (D), *Bc. thetaiotaomicron* VPI-5489 (E), *E. coli* LMG2092 (F), *Bl. coccoides* DSM935 (G), *R. gauvreauii* DSM19829 (H). Average and standard deviation of the percentage of labelled cells obtained for each bacterial species from at least triplicate independent experiments, are presented (I).

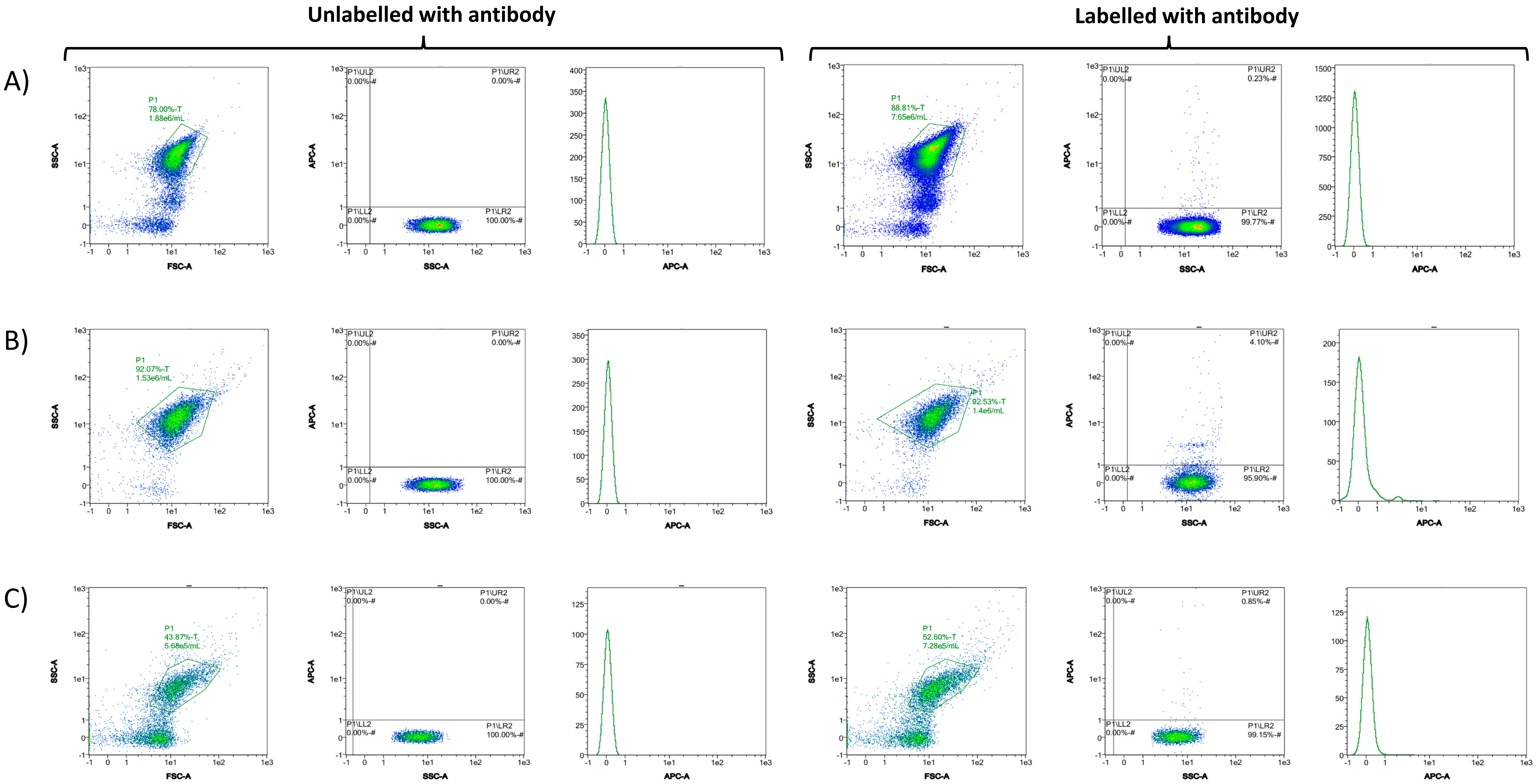

Unlabelled with antibody
Labelled with antibody
A)
P1
78.00%-T
1.88e6/mL
P1\UL2 0.00%-#
P1\UR2 0.00%-#
P1\LL2 0.00%-#
P1\LR2 100.00%-#
P1
88.81%-T
7.65e6/mL
P1\UL2 0.00%-#
P1\UR2 0.23%-#
P1\LL2 0.00%-#
P1\LR2 99.77%-#
B)
P1
92.07%-T
1.53e6/mL
P1\UL2 0.00%-#
P1\UR2 0.00%-#
P1\LL2 0.00%-#
P1\LR2 100.00%-#
P1
92.53%-T
1.4e6/mL
P1\UL2 0.00%-#
P1\UR2 4.10%-#
P1\LL2 0.00%-#
P1\LR2 95.90%-#
C)
P1
43.87%-T
5.68e5/mL
P1\UL2 0.00%-#
P1\UR2 0.00%-#
P1\LL2 0.00%-#
P1\LR2 100.00%-#
P1
52.60%-T
7.28e5/mL
P1\UL2 0.00%-#
P1\UR2 0.85%-#
P1\LL2 0.00%-#
P1\LR2 99.15%-#
FSC-A
SSC-A
APC-A

D)

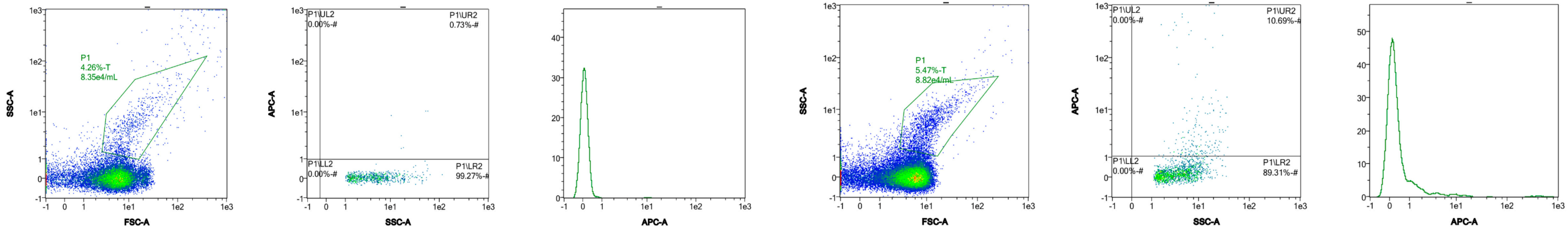

P1
4.26%-T
8.35e4/mL
SSC-A
FSC-A
P1\UL2
0.00%-#
P1\UR2
0.73%-#
P1\LL2
0.00%-#
P1\LR2
99.27%-#
APC-A
SSC-A
APC-A
P1
5.47%-T
8.82e4/mL
SSC-A
FSC-A
P1\UL2
0.00%-#
P1\UR2
10.69%-#
P1\LL2
0.00%-#
P1\LR2
89.31%-#
APC-A
SSC-A
APC-A

E)

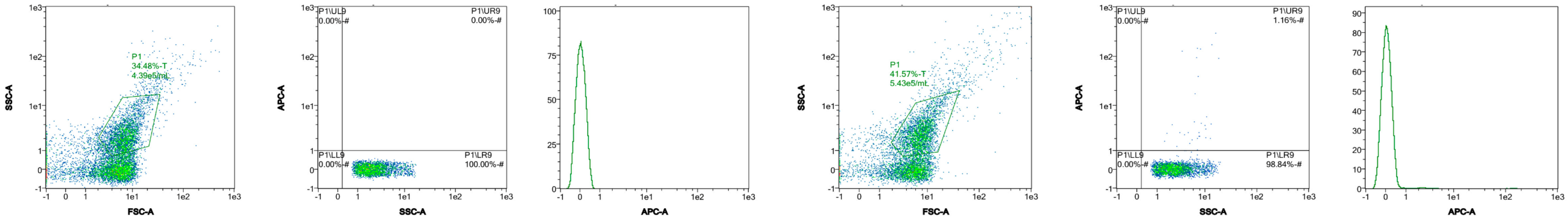

P1
34.48%-T
4.39e5/mL
SSC-A
FSC-A
P1\UL9
0.00%-#
P1\UR9
0.00%-#
P1\LL9
0.00%-#
P1\LR9
100.00%-#
APC-A
SSC-A
APC-A
P1
41.57%-T
5.43e5/mL
SSC-A
FSC-A
P1\UL9
0.00%-#
P1\UR9
1.16%-#
P1\LL9
0.00%-#
P1\LR9
98.84%-#
APC-A
SSC-A
APC-A

F)

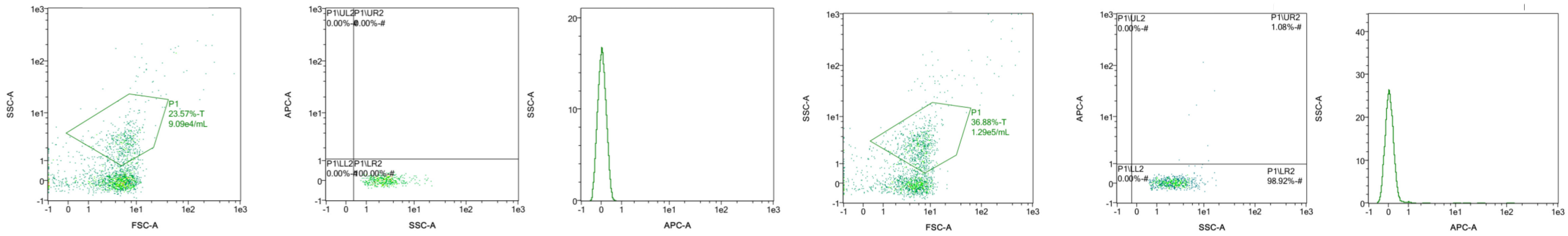

P1
23.57%-T
9.09e4/mL
SSC-A
FSC-A
P1\UL2
0.00%-#
P1\UR2
0.00%-#
P1\LL2
0.00%-#
P1\LR2
100.00%-#
APC-A
SSC-A
SSC-A
APC-A
P1
36.88%-T
1.29e5/mL
SSC-A
FSC-A
P1\UL2
0.00%-#
P1\UR2
1.08%-#
P1\LL2
0.00%-#
P1\LR2
98.92%-#
APC-A
SSC-A
SSC-A
APC-A

G)

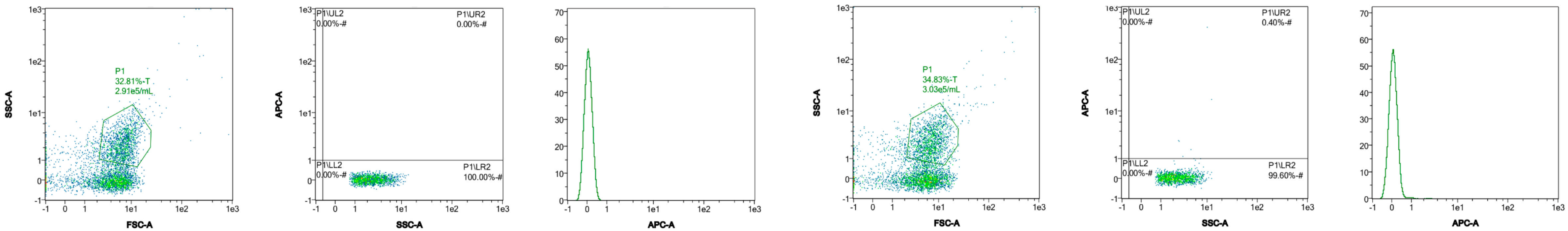

H)

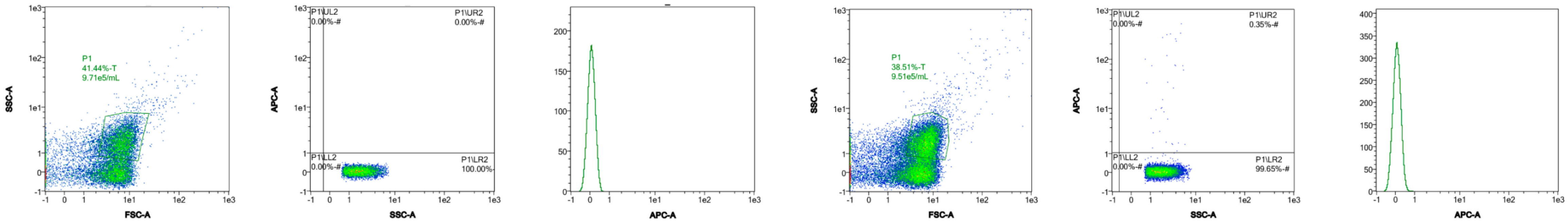

I)

% of events with fluorescence		
	Mean	Standard deviation
***Lactobacillus acidophilus* DSM20079^T**	0.18	0.06
***Bifidobacterium longum* NCIMB8809**	4.72	1.50
***Akkermansia muciniphila* DSM26127**	0.88	0.04
***Collinsella intestinalis* DSM13280**	10.07	1.57
***Bacteroides thetaiotaomicron* VPI-5489**	1.50	0.36
***Escherichia coli* LMG2092**	1.33	0.42
***Blautia coccoides* DSM935**	0.33	0.09
***Ruminococcus gauvreaii* DSM19829**	0.49	0.20

Supplementary figure 3. Representative SDS-PAGE gels showing the protein profiles of *F. prausnitzii* M21, *L. acidophilus* DSM20079^{T}, *B. longum* NCIMB8809, *C. intestinalis* DSM13280, *P. copri* DSM18205, *Bc. thethaiotaomicron* DSM2079, *R. gauvreauii* DSM19829, *Bl. coccoides* DSM935, *A. muciniphila* DSM26127, *E. coli* LMG2092 (A). Immunoreactive bands of the different bacteria labelled with the polyclonal antibody against M21. A secondary anti-rabbit IgA antibody conjugated to HRP was used (B). Minimum triplicate experiments were carried out.

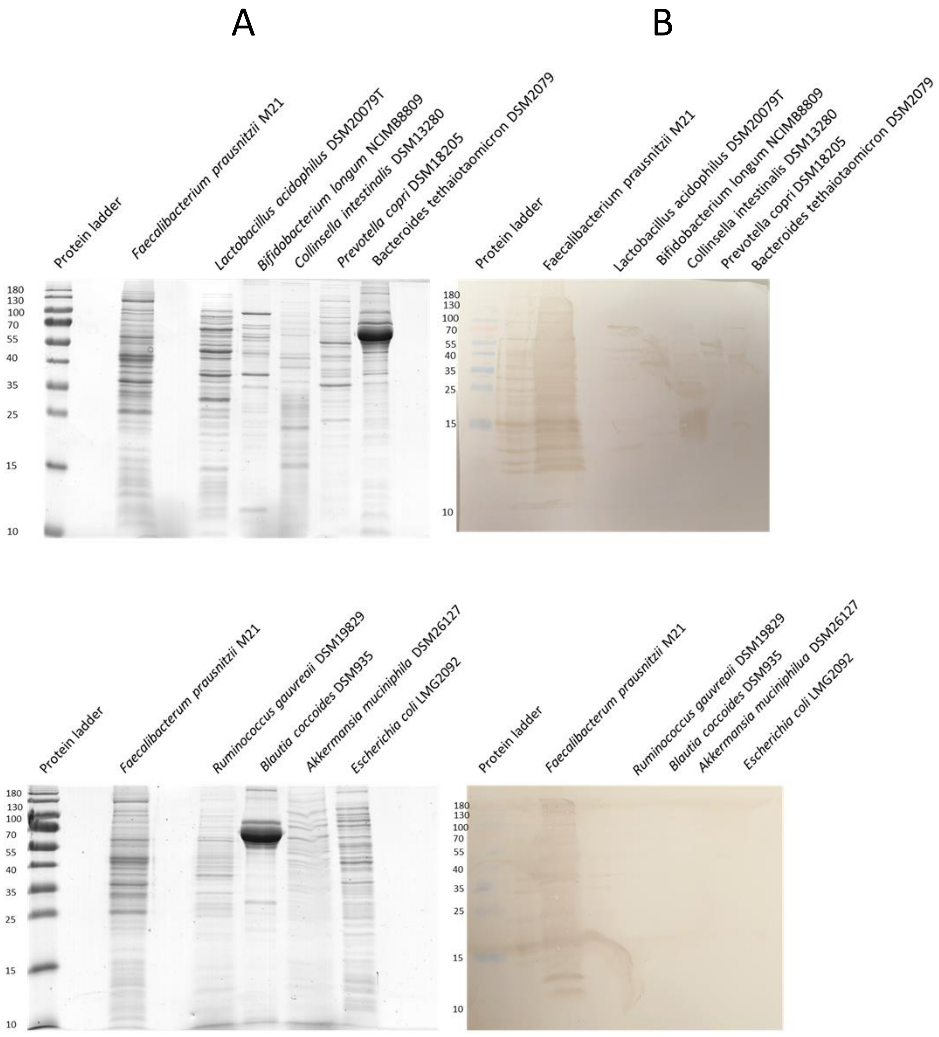

A
B
Protein ladder
Faecalibacterium prausnitzii M21
Lactobacillus acidophilus DSM20079T
Bifidobacterium longum NCIMB8809
Collinsella intestinalis DSM13280
Prevotella copri DSM18205
Bacteroides tethaiotaomicron DSM2079
Ruminococcus gauvreaii DSM19829
Blautia coccoides DSM935
Akkermansia muciniphila DSM26127
Escherichia coli LMG2092
180
130
100
70
55
40
35
25
15
10

Supplementary figure 4. Bar charts showing the integrity and membrane potential of bacteria from microbiotas of healthy donors (A) and Crohn's patients (B). Dispersion diagrams showing the flow cytometry acquisition using SYTO9, PI and DiOC antibodies on the 10 microbiotas from healthy donors (C and D) and the 10 microbiotas from Crohn disease patients (E and F).

A)

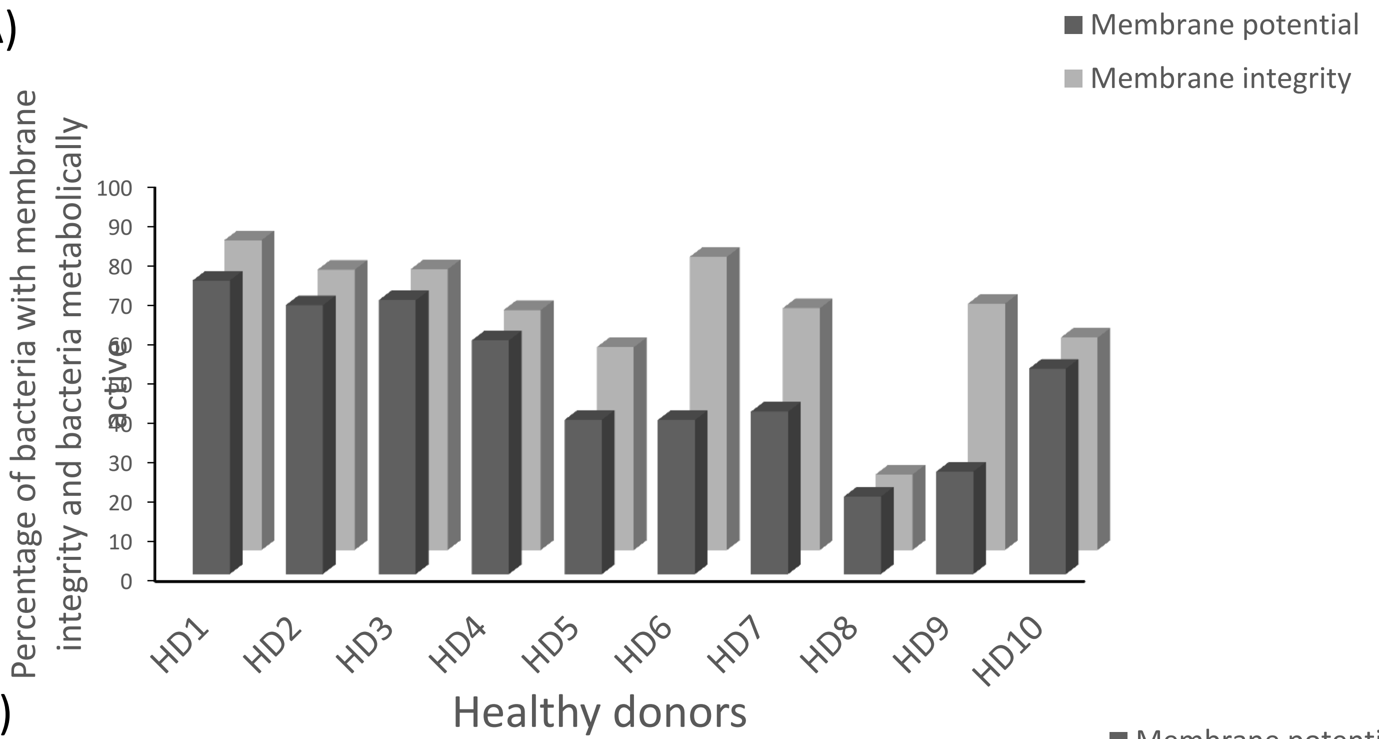
Membrane potential
Membrane integrity
Percentage of bacteria with membrane integrity and bacteria metabolically active
100
90
80
70
60
50
40
30
20
10
0
HD1
HD2
HD3
HD4
HD5
HD6
HD7
HD8
HD9
HD10
Healthy donors

B)

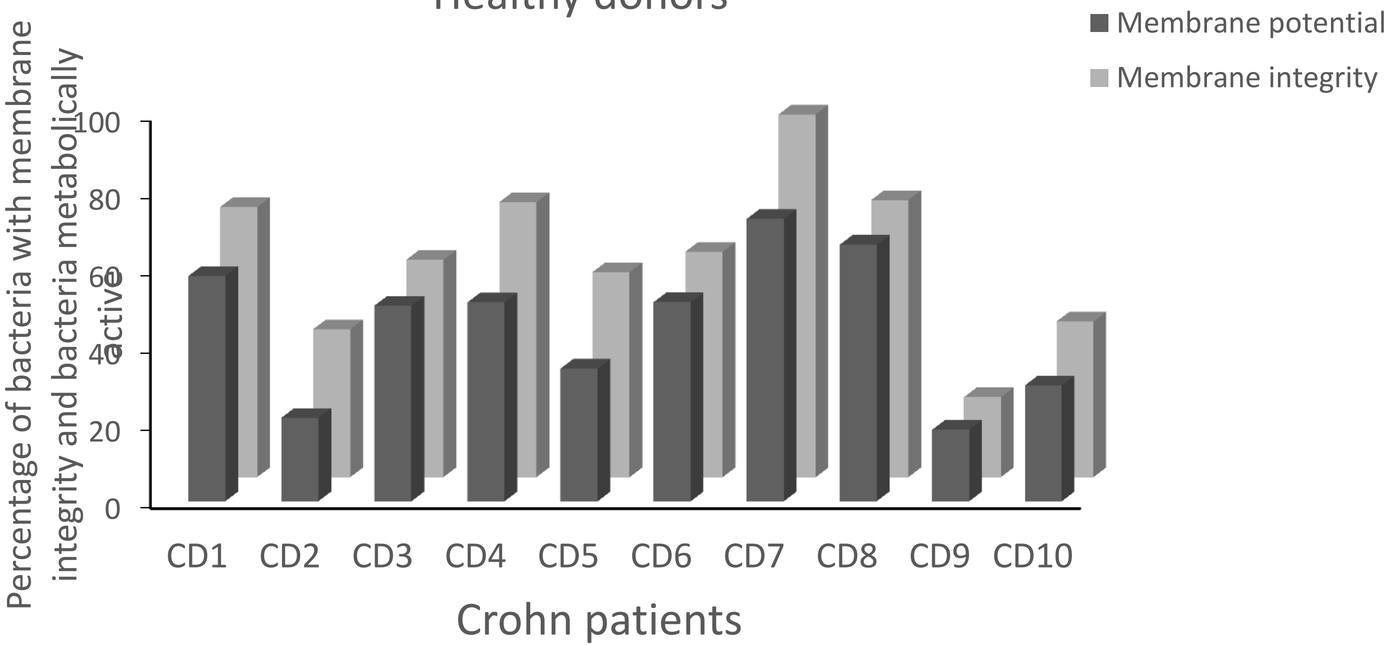
Membrane potential
Membrane integrity
Percentage of bacteria with membrane integrity and bacteria metabolically active
100
80
60
40
20
0
CD1
CD2
CD3
CD4
CD5
CD6
CD7
CD8
CD9
CD10
Crohn patients

Membrane integrity

C

Intact membrane | Compromised membrane

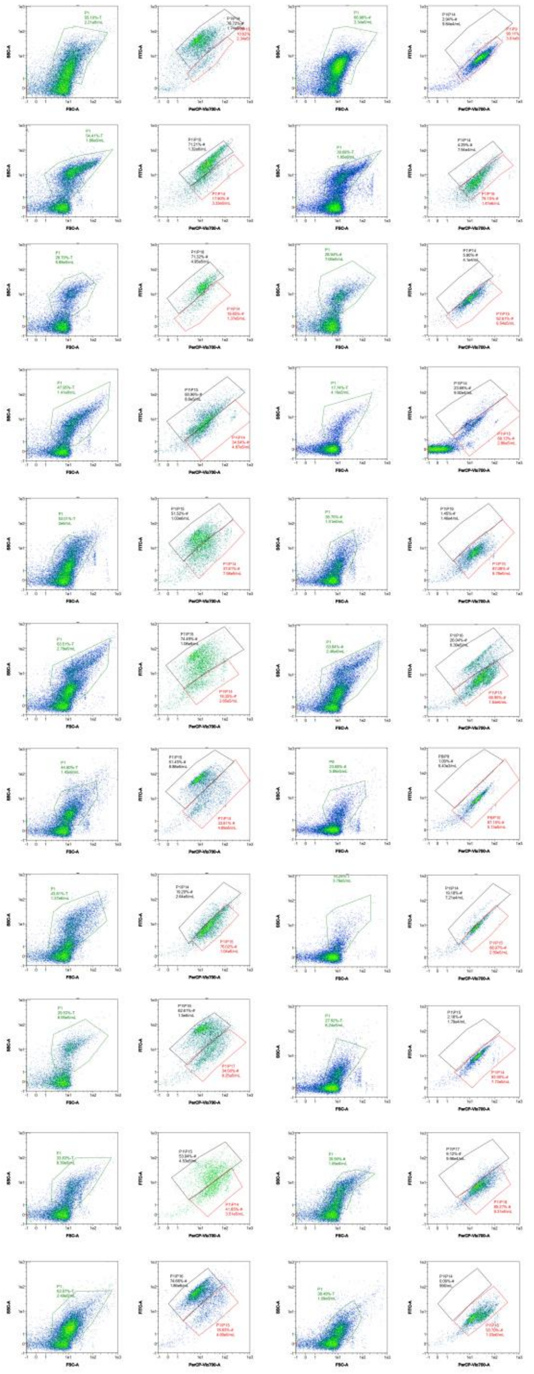

Membrane potential

D

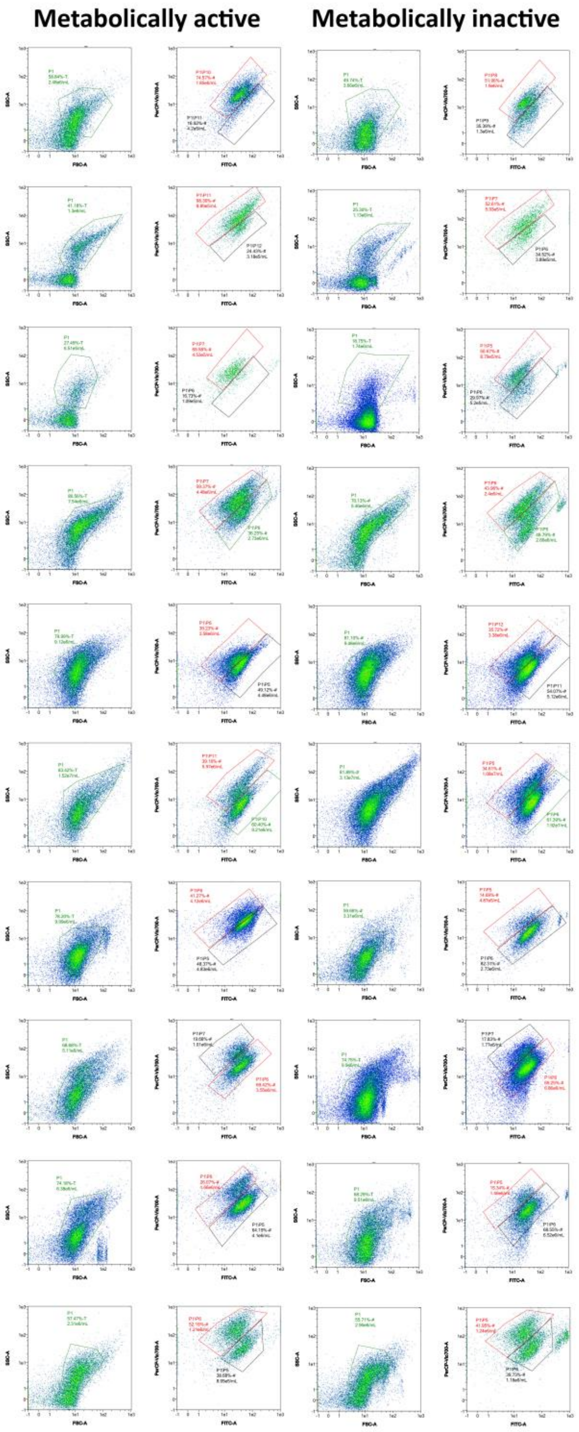

Membrane integrity

E

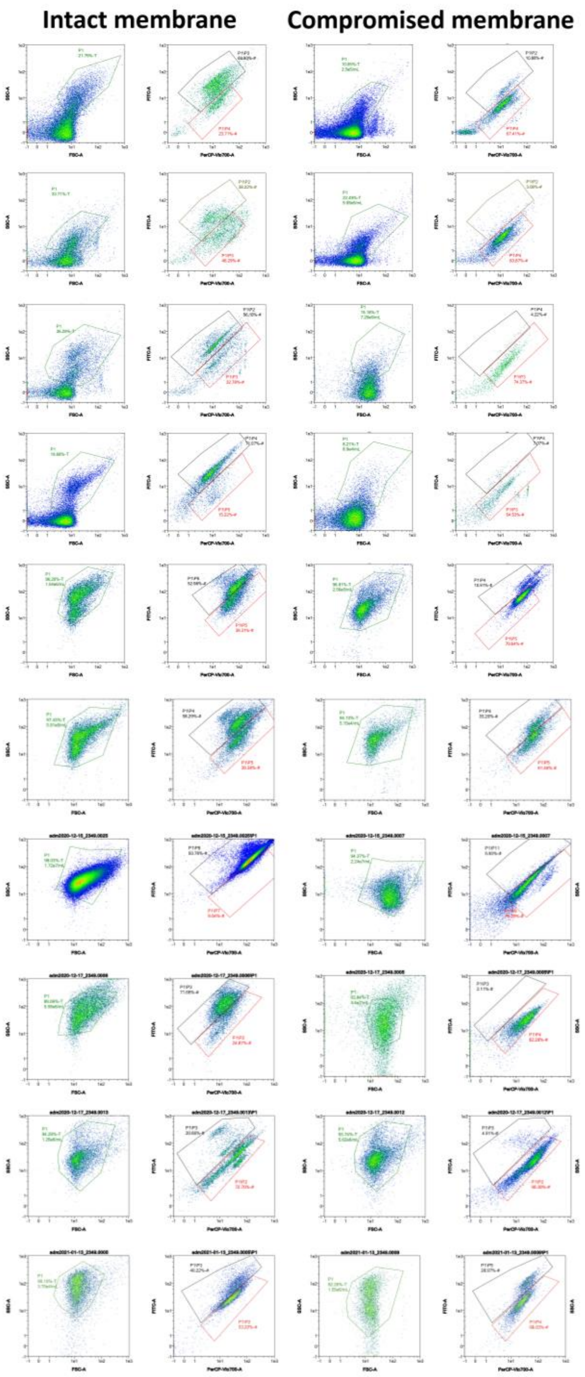

Membrane potential

F

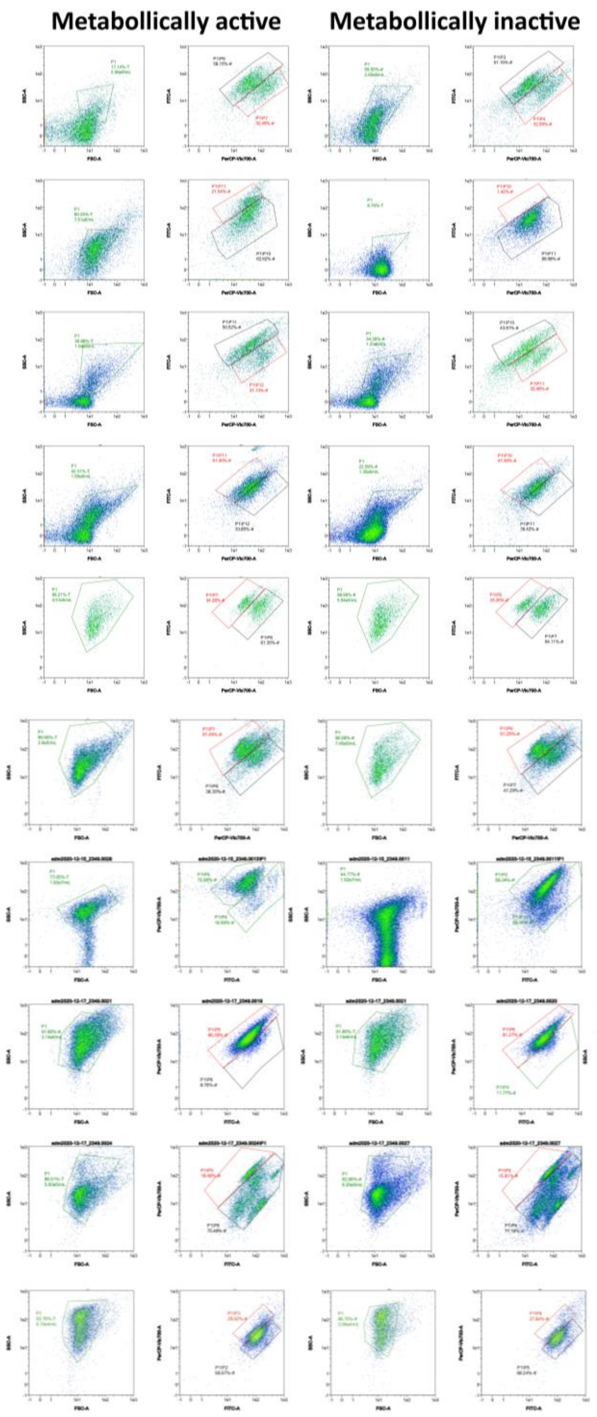

Supplementary figure 5. Bar plots showing the bacterial profile of the HD microbiotas (A) and CD microbiotas (B). Only the twenty most abundant genera are represented and the bars sections representing the relative abundance of *Faecalibacterium* are plotted in red color and highlighted with a * mark to ease its identification on the plots. On top of each bar, the percentage of relative abundance of *F. prausnitzii* sequences is shown for each microbiota. Relative abundance of *F. prausnitzii* in the samples of the four HD microbiotas used in the PBMC coculture experiments and their positive (*Faecalibacterium*-enriched) fractions (C).

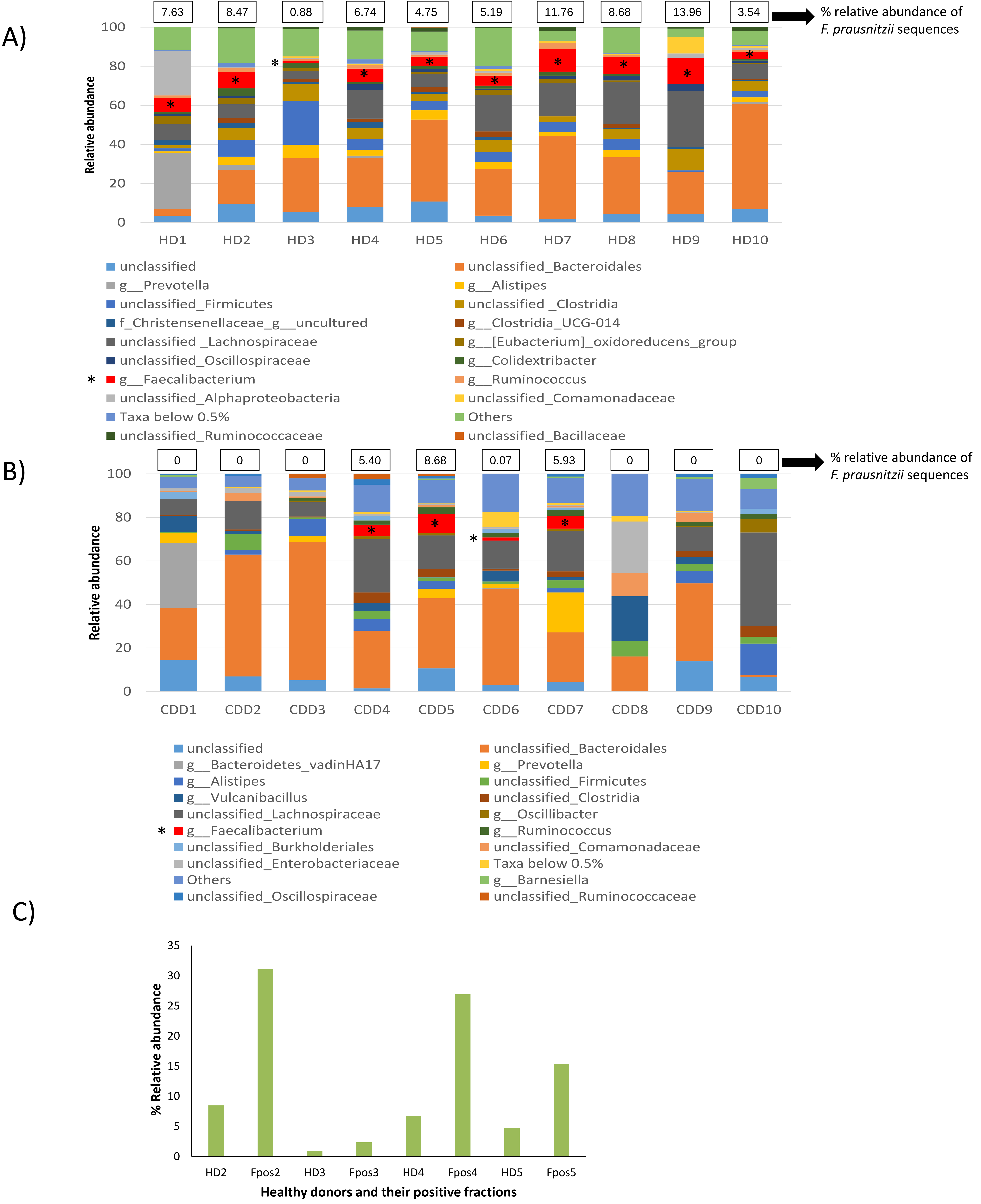
A)
7.63
8.47
0.88
6.74
4.75
5.19
11.76
8.68
13.96
3.54
% relative abundance of
F. prausnitzii sequences
Relative abundance
100
80
60
40
20
0
HD1
HD2
HD3
HD4
HD5
HD6
HD7
HD8
HD9
HD10
unclassified
unclassified_Bacteroidales
g__Prevotella
g__Alistipes
unclassified_Firmicutes
unclassified _Clostridia
f_Christensenellaceae_g__uncultured
g__Clostridia_UCG-014
unclassified _Lachnospiraceae
g__[Eubacterium]_oxidoreducens_group
unclassified_Oscillospiraceae
g__Colidextribacter
* g__Faecalibacterium
g__Ruminococcus
unclassified_Alphaproteobacteria
unclassified_Comamonadaceae
Taxa below 0.5%
Others
unclassified_Ruminococcaceae
unclassified_Bacillaceae
B)
0
0
0
5.40
8.68
0.07
5.93
0
0
0
% relative abundance of
F. prausnitzii sequences
Relative abundance
100
80
60
40
20
0
CDD1
CDD2
CDD3
CDD4
CDD5
CDD6
CDD7
CDD8
CDD9
CDD10
unclassified
unclassified_Bacteroidales
g__Bacteroidetes_vadinHA17
g__Prevotella
g__Alistipes
unclassified_Firmicutes
g__Vulcanibacillus
unclassified_Clostridia
unclassified_Lachnospiraceae
g__Oscillibacter
* g__Faecalibacterium
g__Ruminococcus
unclassified_Burkholderiales
unclassified_Comamonadaceae
unclassified_Enterobacteriaceae
Taxa below 0.5%
Others
g__Barnesiella
unclassified_Oscillospiraceae
unclassified_Ruminococcaceae
C)
% Relative abundance
35
30
25
20
15
10
5
0
HD2
Fpos2
HD3
Fpos3
HD4
Fpos4
HD5
Fpos5
Healthy donors and their positive fractions
